# Computation of Nonparametric, Mixed Effects, Maximum Likelihood, Biosensor Data Based-Estimators for the Distributions of Random Parameters in an Abstract Parabolic Model for the Transdermal Transport of Alcohol

**DOI:** 10.3934/mbe.2023900

**Published:** 2023-11-09

**Authors:** Lernik Asserian, Susan E. Luczak, I. G. Rosen

**Affiliations:** 1 Department of Mathematics, Stanford University, Stanford, CA 94305, USA; 2 Department of Psychology, University of Southern California, Los Angeles, CA 90089, USA; 3 Department of Mathematics, University of Southern California, Los Angeles, CA 90089, USA

**Keywords:** Nonparametric estimation, Mixed effects model, Maximum likelihood estimation, Existence and consistency, Random discrete-time dynamical systems, Random partial differential equations, finite dimensional approximation and convergence, Alcohol biosensor, Transdermal alcohol concentration

## Abstract

The existence and consistency of a maximum likelihood estimator for the joint probability distribution of random parameters in discrete-time abstract parabolic systems are established by taking a nonparametric approach in the context of a mixed effects statistical model using a Prohorov metric framework on a set of feasible measures. A theoretical convergence result for a finite dimensional approximation scheme for computing the maximum likelihood estimator is also established and the efficacy of the approach is demonstrated by applying the scheme to the transdermal transport of alcohol modeled by a random parabolic PDE. Numerical studies included show that the maximum likelihood estimator is statistically consistent, demonstrated by the convergence of the estimated distribution to the “true” distribution in an example involving simulated data. The algorithm developed is then applied to two datasets collected using two different transdermal alcohol biosensors. Using the leave-one-out cross-validation method, we get an estimate for the distribution of the random parameters based on a training set. The input from a test drinking episode is then used to quantify the uncertainty propagated from the random parameters to the output of the model in the form of a 95% error band surrounding the estimated output signal.

## 1. Introduction

In clinical therapy, medical research, and law enforcement, the breathalyzer, developed by Borken-stein based on a redox reaction and Henry’s law [1], is used to measure breath alcohol concentration (BrAC), a surrogate for blood alcohol concentration (BAC). Clinicians and researchers consider it to be reasonably accurate to substitute BrAC for BAC and in general, this continues to be the case across different environmental conditions and across different individuals [1]. Nevertheless, collecting near-continuous BrAC samples accurately (i.e. obtaining a deep lung sample that is not contaminated by any existing alcohol remaining in the mouth) is challenging and often impractical in the field.

Most of the ethanol, the type of alcohol in alcoholic beverages, that enters the human body, is metabolized by the liver into other products that are then excreted. In addition, a portion of ingested ethanol exits the body directly through exhalation and urination [2] and approximately 1% diffuses through the epidermal layer of the skin in the form of perspiration and sweat. The amount of alcohol excreted in this manner is quantified in the form of transdermal alcohol concentration (TAC). TAC has been shown to be largely positively correlated with BrAC and BAC [3]. However, the precise relationship between TAC and BrAC/BAC is complicated due to a number of confounding physiological, technological, and environmental factors including, but not limited to, the skin’s epidermal layer thickness, porosity and tortuosity, the process of vasodilation as observed through blood pressure and flow rate, the underlying technology of the particular sensor being used, and the ambient temperature and humidity.

**Figure 1.**
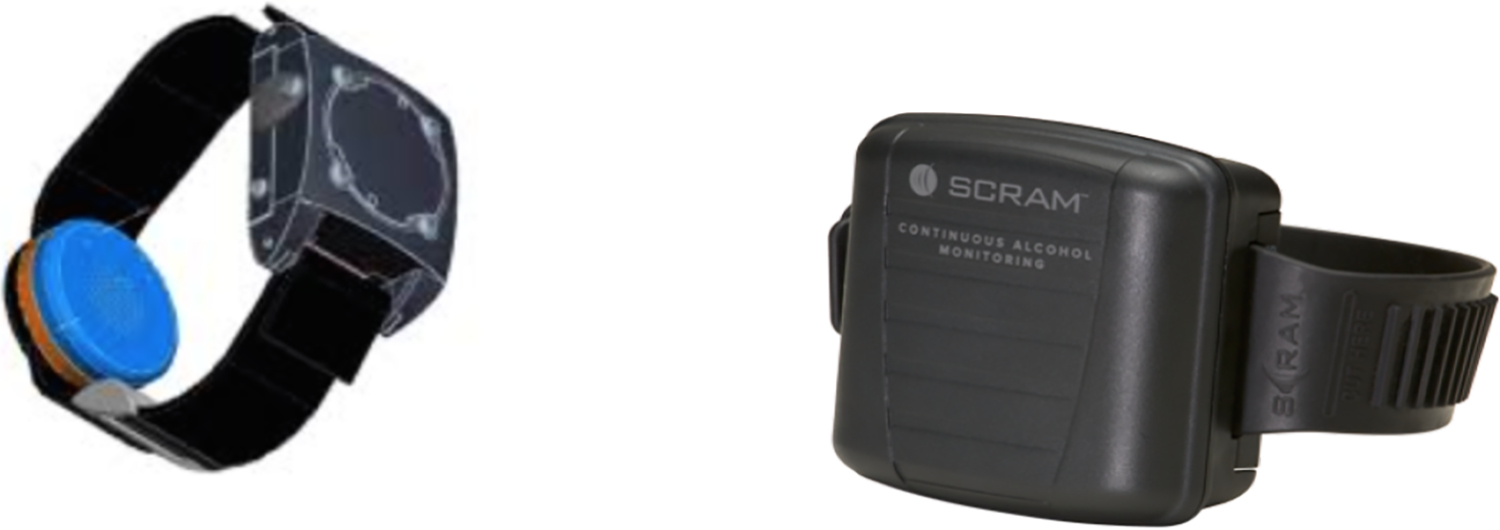
WrisTAS*^™^*7 (left) and SCRAM CAM^®^ (right) transdermal alcohol biosensors

Currently, there are a number of different biosensors based on a variety of analog principles that can measure TAC essentially continuously, passively, unobtrusively, and relatively accurately, and make it available for processing in real time. Some of these devices are already commercially available and more are on the way. Several of these biosensors, like the breathalyzer, rely on relatively standard fuel-cell technology (i.e. converting chemical energy into electricity through redox reactions) to effectively count the number of ethanol molecules that evaporate during perspiration from the epidermal layer of the skin in near-continuous time [4]. Figure 1 shows two of these TAC measuring devices; the WrisTAS*^™^* 7, developed by Giner, Inc. in Waltham, MA and the SCRAM CAM^®^ (Secure Continuous Remote Alcohol Monitor), developed by Alcohol Monitoring Systems, Inc. (AMS) in Littleton, Colorado.

Historically, researchers, clinicians, and the courts have always relied on BrAC or, when available, BAC. Consequently, in order to make TAC biosensors practical and accepted by the alcohol community, reliable and consistent means for converting TAC into equivalent BrAC or BAC must be developed, and this involves challenges that must be dealt with as indicated previously. In the past, our approach to developing a method for converting TAC into BrAC or BAC was based on deterministic methods for estimating parameters in distributed parameter systems such as those described in [5, 6]. Our earlier work along these lines has been reported in, for example, [7–9]. In these treatments, a forward model in the form of a one-dimensional diffusion equation based on Fick’s law [10] with BrAC as the input and TAC as the output is first calibrated (i.e. fit) using BrAC and TAC data collected from the patient or research subject in the clinic or laboratory during what is known as a controlled alcohol challenge. Then, after the same patient or research subject has worn the TAC sensor in the field for an extended period of time (e.g. days, weeks, or even months), the TAC data is downloaded, and the fit forward model is used to deconvolve the BrAC or BAC input from the observed TAC output.

In order to eliminate the calibration process, we developed a population model-based approach wherein the parameters in the model were assumed to be random. Then, rather than fitting the values of the parameters themselves, their distributions were estimated based on BrAC and TAC data from a cohort of individuals (see, for example, [11–17]). We have also developed a number of physics-informed (based on the first principles diffusion-based model given in (1.1)–(1.5) below) data-based machine learning schemes using hidden Markov models, generative adversarial and long-short term memory neural network models for estimating BrAC from biosensor TAC data that are also trained using population data and do not require individual calibration [18–20].

In all of our approaches to the TAC to BAC/BrAC conversion problem, the underlying model was taken to be based on the first principles physics based initial-boundary value problem for a parabolic partial differential equation. This will also be the basic model to which we will direct our efforts in the present treatment. Transformed to be in terms of dimensionless variables, the model is given by

(1.1) $$\frac{\partial x}{\partial t}\left(t,\eta \right)=q_{1}\frac{\partial^{2}x}{\partial \eta^{2}}\left(t,\eta \right),\;0<\eta <1,\;t>0,$$

(1.2) $$q_{1}\frac{\partial x}{\partial \eta }\left(t,0\right)=x\left(t,0\right),\;t>0,$$

(1.3) $$q_{1}\frac{\partial x}{\partial \eta }\left(t,1\right)=q_{2}u\left(t\right),\;t>0,$$

(1.4) $$x\left(0,\eta \right)=x_{0},\;0<\eta <1,$$

(1.5) y(t)=x(t,0),t>0,

where t and η are the temporal and spatial variables, respectively, and x(t,η) indicates the concentration of ethanol in the epidermal layer of the skin at time t and depth η, where η=0 is at the skin surface and η=1 is at the boundary between the epidermal and dermal layers of the skin. The input to the system is u(t), which is the BrAC/BAC at time t, and the output is y(t), which is the TAC at time t. Equation (1.1) represents the transport of ethanol through the epidermal layer of the skin. The boundary conditions (1.2) and (1.3) represent respectively the evaporation of ethanol at the skin surface and the flux of ethanol across the boundary between the epidermal and dermal layers. It is assumed that there is no alcohol in the epidermal layer of the skin at time t=0, so the initial condition (1.4) is $x_{0}(\eta )=0,\;0<\eta <1$. Finally, the output equation (1.5) represents the TAC level measured by the biosensor at the skin surface.

The parameters in the system (1.1)–(1.5) that will be assumed to be random are $q_{1}$ and $q_{2}$, which represent respectively the normalized diffusivity and the normalized flux gain at the boundary between the dermal and epidermal layers. The values or distributions of these parameters are assumed to depend on environmental conditions, the particular sensor being used, and the physiological characteristics of the individual wearing the sensor. The parameter vector is $q=\left(q_{1},q_{2}\right)\in Q$, where Q is assumed to be a compact subset of $R^{+}\times R^{+}$ with metric d.

In population modeling, we can statistically classify the methods as parametric or nonparametric. In the parametric approach, we assume that the general structure of the distribution is known a-priori but with unknown parameters. Then, for example, if we know that the distribution is normal with unknown mean and variance, the estimation problem is to estimate these two unknown parameters. On the other hand; in the nonparametric approach, the structure of the distribution is assumed to be unknown, and the problem is to estimate the distribution itself. In either of these paradigms, different statistical approaches to the estimation problem can be taken. For example, in [15–17], a parametric least squares naive pooled data approach was used, while in [21,22], the approach was nonparametric. In [12], a Bayesian framework was developed, and in the present treatment we consider a mixed effects (see, for example, [23–25]) maximum likelihood based statistical model. In the mixed effects model, it is assumed that observations are specific to a single individual plus a random error. The mixed effects model is a combination of the fixed-effects model, which describes the characteristics for an average individual in the population, and the random-effects model, which describes the inter-individual variability [26]. An overview of these different statistical approaches in the context of pharmacokinetics can be found in [27].

While TAC and BrAC are related, they are not precisely equivalent in terms of quantification. BrAC serves as a reliable indicator of BAC, offering valuable quantitative insights into the immediate impact of alcohol on judgment, motor skills, and cognition. On the contrary, TAC lags behind BrAC because it must pass through the skin before measurement. TAC levels exhibit variability among individuals, devices, and across drinking episodes, in contrast to the relative consistency of BrAC across different people and conditions. Consequently, interpreting TAC in relation to real-time blood alcohol levels becomes challenging, limiting its usefulness as a quantitative alcohol measure. Therefore, having a method to estimate BrAC from TAC would enhance its practicality for researchers, healthcare professionals, and individuals seeking meaningful and time-sensitive information about their alcohol levels. The inverse problem of estimating the input BrAC signal, u, to the system, (1.1)–(1.5), based on observations of the TAC output signal, y, is the central focus of our team’s research effort. In our treatment here, we consider only the problem of estimating the distribution of the random parameters in the population model and how the uncertainty in the parameters propagates through the system of equations (1.1)–(1.5) to the TAC output, y. Once the distributions of the random parameters in the the model (1.1)–(1.5) have been estimated, we have previously developed (and continue to investigate) a number of deconvolution or inverse filtering methods that can then be used to obtain an estimate for the BrAC signal along with a credible or error band that result from the uncertainty in the model parameters as described by their estimated distributions. We have looked at a number of approaches including frequency domain, Bayesian, linear-quadratic Gaussian compensator, maximum likelihood, ARMA, nonlinear least squares based techniques [11, 28–31].

In addition to the work of our group on TAC to BAC/BrAC conversion cited above, other researchers have also been looking at this problem and have tried a number of different approaches. For example, in [32,33], a more traditional approach based on standard linear regression techniques is developed and discussed. A number of ideas from the machine learning literature have also been considered. In [34], a scheme based on random forests is used to recover BrAC from TAC, and in our group, there are the machine learning based treatments cited earlier [18–20].

An outline of the remainder of the paper is as follows. In Section 2, we provide a summary of the Prohorov metric on the set of probability measures as it was used by Banks and his co-authors in [21]. In Section 3, we define our mathematical model in the form of a random discrete-time dynamical system and we define the maximum likelihood estimator for the distribution of the random parameters. In Section 4, we establish the existence and consistency of the maximum likelihood estimator for the distribution of the random parameters, while in Section 5, we demonstrate the convergence of finite dimensional approximations for our estimator. In Section 6, we summarize results for abstract parabolic systems, their finite dimensional approximation, and an associated convergence theory. In Section 7, the application of our scheme to the transdermal transport of alcohol is presented and discussed. This includes numerical studies for two examples, one involving simulated data and the other, actual data collected in the laboratory of one of the co-authors, Dr. Susan Luczak, in the Department of Psychology at University of Southern California (USC). For the simulated data example in Section 7.1, we are able to observe the convergence of the estimated distribution of the random parameter vector $q=\left(q_{1},q_{2}\right)$ to the “true” distribution as the number of drinking episodes increases, as the number of Dirac measure nodes increases, and as the level of discretization in the finite dimensional approximations increases. In the actual data example discussed in Section 7.2, we apply the leave-one-out cross-validation (LOOCV) method by first estimating the distribution of the parameter vector q using a training set, and then estimating the TAC output using the estimated distribution and the BrAC input of a testing episode.

## 2. Prohorov Metric Framework

Banks and his co-authors developed a framework for estimation of the probability measure for random parameters in continuous-time dynamical systems based on the Prohorov metric [21]. Here, we summarize the Prohorov metric and its properties.

Let Q be a Hausdorff metric space with metric d. Define

$$C_{b}\left(Q\right)=\left\{f:Q\to \mathbb{R}|f\;\text{is}\;\text{bounded}\;\text{and}\;\text{continuous}\;\right\},$$

and given any probability measure P∈𝒫(Q), where 𝒫(Q) denotes the set of all probability measures defined on $\Sigma_{Q}$, the Borel sigma algebra on Q, and some ε>0, an ε-neighborhood of P is defined by

$$B_{\varepsilon }\left(P\right)=\left\{\tilde{P}|\left|\int_{Q}f\left(q\right)d\tilde{P}\left(q\right)-\int_{Q}f\left(q\right)dP\left(q\right)\;\right|<\varepsilon ,\;\text{for}\;\text{all}\;f\in C_{b}\left(Q\right)\right\}.$$

Let $E\in \Sigma_{Q}$, and define the ε-neighborhood of E by

$$E^{\varepsilon }=\{\tilde{q}\in Q|d\left(\tilde{q},E\right)<\varepsilon \}=\left\{\tilde{q}\in Q|\underset{q\in E}{\text{inf}\;}d\left(q,\tilde{q}\right)<\varepsilon \right\}.$$

Given two probability measures, P and $\tilde{P}$ in 𝒫(Q), the Prohorov metric ρ on 𝒫(Q)×𝒫(Q) is defined such that

$$\tilde{P}\in B_{\varepsilon }\left(P\right)\Leftrightarrow \rho \left(P,\tilde{P}\right)<\varepsilon ,$$

where

$$\rho \left(P,\tilde{P}\right)=\text{inf}\left\{\varepsilon >0|\tilde{P}\left(E\right)\leq P\left(E^{\varepsilon }\right)+\varepsilon \;\text{and}\;P\left(E\right)\leq \tilde{P}\left(E^{\varepsilon }\right)+\varepsilon ,\;\text{for}\;\text{all}\;E\in \Sigma_{Q}\right\}.$$

It can be shown that (𝒫(Q),ρ) is a metric space. Also, the Prohorov metric metrizes the weak convergence of measures, i.e. given a sequence of measures $P_{M}\in 𝒫(Q)$, for all M=1,
2,…, and P∈𝒫(Q),

$$P_{M}\overset{w^{*}}{\to }P\Leftrightarrow \rho \left(P_{M},P\right)\to 0.$$

It is important to note that the weak* topology and the weak topology are equivalent on the space of probability measures.

For some $n_{q},\;Q\subseteq R^{n_{q}}$ and P∈𝒫(Q), consider the random vector $X:Q\to R^{n_{q}}$ on the probability space $\left(Q,\Sigma_{Q},P\right)$ given by X(q)=q for q∈Q. The cumulative distribution function for X is given by $F_{X}\left(q_{1},\ldots ,q_{n}\right)=P\left(X\in \times_{\ell =1}^{n_{q}}\left(-\infty ,q_{\ell }\right]\right)=P\left(\times_{\ell =1}^{n_{q}}\left\{\left(-\infty ,q_{\ell }\right]\cap Q\right\}\right)$. In this case, it follows that if $P_{M},\;P_{0}\in (𝒫(Q),\rho )$, for M=1,
2,…, then $\rho \left(P_{M},P_{0}\right)\to 0$ if and only if $F_{X_{M}}\to F_{X_{0}}$ at all points of continuity of $F_{X_{0}}$. Consequently, Prohorov metric convergence and weak and weak* convergence in 𝒫(Q) are also referred to as convergence in distribution.

If $q_{1},q_{2}\in Q$, then $\rho \left(\delta_{q_{1}},\delta_{q_{2}}\right)=\text{min}\left\{d\left(q_{1},q_{2}\right),1\right\}$, where $\delta_{q_{j}}\in D=\left\{\delta_{q}|q\in Q\right\}$, the space of Dirac measures on Q, where for all $E\in \Sigma_{Q}$,

$$\delta_{q}\left(E\right)=\{\begin{array}{ll} 1 & \text{if}\;q\in E\\ 0 & \text{if}\;q\notin E \end{array}.$$

The metric space (𝒫(Q),ρ) is separable if and only if the metric space (Q,d) is separable. The sequence ${\left\{q_{j}\right\}}_{j=1}^{\infty }$ is Cauchy in (Q,d) if and only if the sequence ${\left\{\delta_{q_{j}}\right\}}_{j=1}^{\infty }$ is Cauchy in (𝒫(Q),ρ). We also have (Q,d) is complete if and only if (𝒫(Q),ρ) is complete, and (Q,d) is compact if and only if (𝒫(Q),ρ) is compact. The details and proofs can be found in [21].

Assume the metric space (Q,d) is separable and let $Q_{d}={\left\{q_{j}\right\}}_{j=1}^{\infty }$ be a countable dense subset of Q. Define the dense (see [21]) subset of 𝒫(Q), ${\tilde{𝒫}}_{d}(Q)$, as

(2.1) $${\tilde{𝒫}}_{d}\left(Q\right)=\left\{P\in 𝒫\left(Q\right)|P=\overset{M}{\underset{j=1}{\sum }}p_{j}\delta_{q_{j}},q_{j}\in Q_{d},M\in \mathbb{N},p_{j}\in \left[0,1\right]\cap \mathbb{Q},\overset{M}{\underset{j=1}{\sum }}p_{j}=1\right\},$$

the collection of all convex combinations of Dirac measures on Q with rational weights $p_{j}$ at nodes $q_{j}\in Q_{d}$, and for each M∈N let

$${𝒫}_{M}\left(Q\right)=\left\{P\in {\tilde{𝒫}}_{d}\left(Q\right)|P=\overset{M}{\underset{j=1}{\sum }}p_{j}\delta_{q_{j}},q_{j}\in {\left\{q_{j}\right\}}_{j=1}^{M}\right\}.$$

## 3. The Mathematical Model

Consider the following discrete-time mathematical model for the $i^{\text{th}}$ subject at time-step k

$$x_{k,i}\left(q_{i}\right)=g_{k-1}\left(x_{k-1,i}\left(q_{i}\right),u_{k-1,i};q_{i}\right),\;k=1,\ldots ,n_{i},\;i=1,\ldots ,m,\;x_{0,i}=\phi_{0,i},\;i=1,\ldots ,m,$$

where $q_{i}$ is the $i^{th}$ subject’s parameter vector in Q, denoting the set of admissible parameters, $g_{k-1}:\;ℋ\times R^{v}\times Q\to ℋ,\;ℋ$ is in general an infinite dimensional Hilbert space, and $u_{k-1,i}\in R^{v}$ is the input. The output is given by

$$y_{k,i}\left(q_{i}\right)=h_{k}\left(x_{k,i}\left(q_{i}\right),\phi_{0,i},u_{k,i};q_{i}\right),\;k=1,\ldots ,n_{i},\;i=1,\ldots ,m,$$

where $h_{k}:ℋ\times ℋ\times R^{v}\times Q\to R$.

For the mixed effects model, we define

(3.1) $$Y_{k,i}=y_{k,i}\left(q_{i}\right)+e_{k,i},\;k=1,\ldots ,n_{i},\;i=1,\ldots ,m,$$

where for each $i=1, 2,\ldots ,m,e_{k,i}$ are independent and identically distributed (i.i.d.) with mean 0, variance $\sigma^{2}$, and $e_{k,i}\sim \varphi ,k=1, 2,\ldots ,n_{i}$, where φ is a density with respect to a sigma finite measure μ on R and assumed to be continuous on R. We assume that the random vectors $\left[e_{1,i},\ldots ,e_{n_{i},i}\right]$ are independent with respect to i,i=1,
2,…,m; that is, the error is independent across individuals and conditionally independent within individuals (i.e. given $q_{i}$). For each i=1,
2,…,m, let $Y_{i}={\left[Y_{1,i},Y_{2,i},\ldots ,Y_{n_{i},i}\right]}^{T},\;y_{i}\left(q_{i}\right)={\left[y_{1,i}\left(q_{i}\right),y_{2,i}\left(q_{i}\right),\ldots ,y_{n_{i},i}\left(q_{i}\right)\right]}^{T},e_{i}={\left[e_{1,i},e_{2,i},\ldots ,e_{n_{i},i}\right]}^{T}$ and rewrite (3.1) as

$$Y_{i}=y_{i}\left(q_{i}\right)+e_{i},\;i=1,\ldots ,m.$$

Then, for $i=1, 2,\ldots ,m,Y_{i}$ are independent with $Y_{i}\sim f_{i}\left(\cdot ;q_{i}\right):R^{n_{i}}\to R$, where

(3.2) $$f_{i}\left(v;q_{i}\right)=\overset{n_{i}}{\underset{k=1}{\prod }}\varphi \left(v_{k}-y_{k,i}\left(q_{i}\right)\right),\;i=1,\ldots ,m,$$

where $v={\left[v_{1},v_{2},\ldots ,v_{n_{i}}\right]}^{T}\in R^{n_{i}}$. Let P∈𝒫(Q) denote a probability measure on $\Sigma_{Q}$, where 𝒫(Q) denotes the set of all probability measures defined on $\Sigma_{Q}$, and let $P_{0}\in 𝒫(Q)$ be the “true” distribution of the random vector $q_{i}$. The goal is to find an estimate of $P_{0}$. In order to generate an estimator for $P_{0}$, and establish theoretical results and computational tools, we use the nonparametric maximum likelihood (NPML) approach, introduced by Lindsay and Mallet in [35,36], using the Prohorov metric-based framework on 𝒫(Q), introduced by Banks and his co-authors in [21], summarized in Section 2.

For P∈𝒫(Q) and i=1,
2,…,m, let

(3.3) $${ℒ}_{i}\left(P;Y_{i}\right)=\int_{Q}f_{i}\left(Y_{i};q_{i}\right)dP\left(q_{i}\right)\;=\int_{Q}\overset{n_{i}}{\underset{k=1}{\prod }}\varphi \left(Y_{k,i}-h_{k}\left(x_{k,i}\left(q_{i}\right),\phi_{0,i},u_{k,i};q_{i}\right)\right)dP\left(q_{i}\right)$$

be the contribution of the $i^{\text{th}\;}$ subject to the likelihood function

(3.4) $${ℒ}_{n,m}\left(P;Y\right)=\overset{m}{\underset{i=1}{\prod }}{ℒ}_{i}\left(P;Y_{i}\right)\;=\overset{m}{\underset{i=1}{\prod }}\int_{Q}f_{i}\left(Y_{i};q_{i}\right)dP\left(q_{i}\right)\;=\overset{m}{\underset{i=1}{\prod }}\int_{Q}\overset{n_{i}}{\underset{k=1}{\prod }}\varphi \left(Y_{k,i}-h_{k}\left(x_{k,i}\left(q_{i}\right),\phi_{0,i},u_{k,i};q_{i}\right)\right)dP\left(q_{i}\right),$$

where $n={\left\{n_{i}\right\}}_{i=1}^{m}$ and $Y={\left\{Y_{i}\right\}}_{i=1}^{m}$. The goal is to find P that maximizes the likelihood function.

Define the estimator

(3.5) $$P_{n,m}=\text{arg}\underset{P\in 𝒫\left(Q\right)}{\text{max}}{ℒ}_{n,m}\left(P;Y\right)\;=\text{arg}\underset{P\in 𝒫\left(Q\right)}{\text{max}}\overset{m}{\underset{i=1}{\prod }}\int_{Q}f_{i}\left(Y_{i};q_{i}\right)dP\left(q_{i}\right)\;=\text{arg}\underset{P\in 𝒫\left(Q\right)}{\text{max}}\overset{m}{\underset{i=1}{\prod }}\underset{Q}{\int }\overset{n_{i}}{\underset{k=1}{\prod }}\varphi \left(Y_{k,i}-h_{k}\left(x_{k,i}\left(q_{i}\right),\phi_{0,i},u_{k,i};q_{i}\right)\right)dP\left(q_{i}\right).$$

Let ${\overset{ˆ}{𝒴}}_{k,i}$ be realizations of the random variables $Y_{k,i}$, and define

(3.6) $${\hat{P}}_{n,m}=\text{arg}\underset{P\in 𝒫\left(Q\right)}{\text{max}}{ℒ}_{n,m}\left(P;\hat{𝓨}\right)\;=\text{arg}\underset{P\in 𝒫\left(Q\right)}{\text{max}}\overset{m}{\underset{i=1}{\prod }}\int_{Q}f_{i}\left({\hat{𝓨}}_{i};q_{i}\right)dP\left(q_{i}\right)\;=\text{arg}\underset{P\in 𝒫\left(Q\right)}{\text{max}}\overset{m}{\underset{i=1}{\prod }}\int_{Q}\overset{n_{i}}{\underset{k=1}{\prod }}\varphi \left({\hat{𝒴}}_{k,i}-h_{k}\left(x_{k,i}\left(q_{i}\right),\phi_{0,i},u_{k,i};q_{i}\right)\right)dP\left(q_{i}\right),$$

where $\overset{ˆ}{𝓨}={\left\{{\overset{ˆ}{𝓨}}_{i}\right\}}_{i=1}^{m}$, with ${\overset{ˆ}{𝓨}}_{i}={\left[{\overset{ˆ}{𝒴}}_{1,i},{\overset{ˆ}{𝒴}}_{2,i},\ldots ,{\overset{ˆ}{𝒴}}_{n_{i},i}\right]}^{T}$.

The results of Lindsay and Mallet in [35,36] states that the maximum likelihood estimator ${\overset{ˆ}{P}}_{n,m}$ can be found in the class of discrete distributions with at most m support points; that is, ${\overset{ˆ}{P}}_{n,m}\in {𝒫}_{M}(Q)$, where M≤m. So, we define our approximating estimator over the set ${𝒫}_{M}(Q)$ where M denotes the number of nodes, ${\left\{q_{j}\right\}}_{j=1}^{M}$. As a result, the optimization is over a finite set of parameters, being the rational weights ${\left\{p_{j}\right\}}_{j=1}^{M}$.

Also, we cannot exactly compute the maximum likelihood estimator, ${\overset{ˆ}{P}}_{n,m}$, since $y_{k,i}$ must be approximated numerically by $y_{k,i}^{N}$ using a Galerkin numerical scheme with N denoting the level of discretization. Thus, our approximating estimator is

(3.7) $${\hat{P}}_{n,m,M}^{N}=\text{arg}\underset{P\in {𝒫}_{M}\left(Q\right)}{\text{max}}{ℒ}_{n,m}^{N}\left(P;\hat{𝓨}\right)\;=\text{arg}\underset{P\in {𝒫}_{M}\left(Q\right)}{\text{max}}\overset{m}{\underset{i=1}{\prod }}\int_{Q}f_{i}^{N}\left({\hat{𝓨}}_{i};q_{i}\right)dP\left(q_{i}\right)\;=\text{arg}\underset{P\in {𝒫}_{M}\left(Q\right)}{\text{max}}\overset{m}{\underset{i=1}{\prod }}\int_{Q}\overset{n_{i}}{\underset{k=1}{\prod }}\varphi \left({\hat{𝒴}}_{k,i}-h_{k}\left(x_{k,i}^{N}\left(q_{i}\right),\phi_{0,i},u_{k,i};q_{i}\right)\right)dP\left(q_{i}\right).$$

## 4. Existence and Consistency of the Maximum Likelihood Estimator

In [35], the existence and uniqueness of a maximum likelihood estimator of a mixing distribution using the geometry of mixture likelihoods was established. Similarly, in [36], the existence and uniqueness of the maximum likelihood estimator for the distribution of the parameters of a random coefficient regression model was established. Here we provide an existence argument based on the maximization of a continuous function over a compact set.

The following theorem establishes the existence of the estimator ${\overset{ˆ}{P}}_{n,m}$ in (3.7), obtained from the realizations $\left\{{\overset{ˆ}{𝒴}}_{k,i}\right\},\;k=1,\ldots ,n_{i},i=1,\ldots ,m$ of the random variables $\left\{Y_{k,i}\right\},\;k=1,\ldots ,n_{i},\;i=1,\ldots ,m$. This is sufficient for establishing the existence of the maximum likelihood estimator $P_{n,m}$ in (3.5).

**Theorem 4.1.** *For* i=1,
2,…,m, *let* ${ℒ}_{i}$ *be given by* equation (3.3), *and let* ${ℒ}_{n,m}(P;\overset{ˆ}{𝓨})$ *be given by* equation (3.4) *where* $\overset{ˆ}{𝓨}={\left\{{\overset{ˆ}{𝓨}}_{i}\right\}}_{i=1}^{m}$, *with* ${\hat{𝓨}}_{i}={\left[{\hat{𝒴}}_{1,i},{\hat{𝒴}}_{2,i},\ldots ,{\hat{𝒴}}_{n_{i},i}\right]}^{T}$. *Assume that for each ${\overset{ˆ}{𝓨}}_{i}$*, *we have a continuous function ${ℒ}_{i}\left(.;{\overset{ˆ}{𝓨}}_{i}\right):𝒫(Q)\to R$*, *and also for each* P∈𝒫(Q) *we have a measurable function* ${ℒ}_{i}(P;.):R^{n_{i}}\to R$. *Then there exists a measurable function ${\overset{ˆ}{P}}_{n,m}:\prod_{i=1}^{m}\;R^{n_{i}}\to 𝒫(Q)$ such that*

$${ℒ}_{n,m}\left({\hat{P}}_{n,m};\hat{𝓨}\right)=\underset{P\in 𝒫\left(Q\right)}{\text{sup}}{ℒ}_{n,m}\left(P;\hat{𝓨}\right).$$

*Proof*. The theorem can be proven in a similar way as in [21] with the difference that we are taking the sup (instead of the inf) of a continuous function over a compact set. □

In order to establish the consistency of the maximum likelihood estimator $P_{n,m}$, we show that $\rho \left(P_{n,m},P_{0}\right)$ converges almost surely to zero. We do this by applying a theorem by Kiefer and Wolfowitz in [37], establishing that the nonparametric maximum likelihood approach is statistically consistent. In other words, as the number of subjects, m, gets larger, the estimator $P_{n,m}$ converges in probability to $P_{0}$, the “true” distribution, in the sense of the Prohorov metric, or weakly, or in distribution. Here, we have set up our problem in a way that makes establishing the consistency a straightforward application of the consistency result in [37].

**Theorem 4.2.** *For each* i=1,…,m, *assume that the map* $q_{i}\mapsto f_{i}\left(v;q_{i}\right)$ *from* Q *into* R *is continuous for each* $v\in R^{n_{i}}$, *and* $f_{i}\left(v;q_{i}\right)$ *is measurable in* v *for any* $q_{i}\in Q$, *where* $f_{i}$ *is given by* (3.2). *Assume further that* $P_{0}$ *is identifiable; that is, for* $P_{1}\in 𝒫(Q)$ *with* $P_{1}\neq P_{0}$, *we have*

$$\overset{m}{\underset{i=1}{\prod }}\int_{0}^{z_{i}}\int_{Q}\overset{n_{i}}{\underset{k=1}{\prod }}\varphi \left(z_{k}-h_{k}\left(x_{k,i}\left(q_{i}\right),\phi_{0,i},u_{k,i};q_{i}\right)\right)dP_{1}\left(q_{i}\right)d\mu^{n_{i}}\;\neq \overset{m}{\underset{i=1}{\prod }}\int_{0}^{z_{i}}\int_{Q}\overset{n_{i}}{\underset{k=1}{\prod }}\varphi \left(z_{k}-h_{k}\left(x_{k,i}\left(q_{i}\right),\phi_{0,i},u_{k,i};q_{i}\right)\right)dP_{0}\left(q_{i}\right)d\mu^{n_{i}},$$

*for at least one* $z={\left[z_{1}^{T},\ldots ,z_{m}^{T}\right]}^{T}\in R^{\sum_{i=1}^{m}\;n_{i}}$, *where for* $i=1, 2,\ldots ,m,\;z_{i}={\left[z_{1},\ldots ,z_{n_{i}}\right]}^{T}\in R^{n_{i}}$, *and the technical integrability assumption holds; that is, for any* P∈𝒫(Q),

$$\underset{\varepsilon ↓0}{\text{lim}}E_{P_{0}}{\left[\text{log}\frac{\underset{\tilde{P}\in B_{\varepsilon }\left(P\right)}{\text{sup}}{ℒ}_{i}\left(\tilde{P};Y_{i}\right)}{{ℒ}_{i}\left(P_{0};Y_{i}\right)}\right]}^{+}<\infty ,$$

*where* ${ℒ}_{i}$ *is given by* equation (3.3).* Then, as* $m\to \infty ,\;\rho \left(P_{n,m},P_{0}\right)\to 0$ *almost surely (i.e with probability 1) and therefore in probability as well*.

*Proof*. The assumptions we have made in the previous section and in the statement of the theorem are sufficient to argue that assumptions 1–5 in [37] are satisfied. The conclusion of the consistency result in [37] is that the cumulative distribution functions, $F_{n,m}$, corresponding to $P_{n,m}$ converge almost surely to the cumulative distribution function $F_{0}$ corresponding to $P_{0}$ at every point of continuity of $F_{0}$. It follows that $\rho \left(P_{n,m},P_{0}\right)\to 0$ almost surely (i.e with probability 1); thus, in probability as well, as m→∞, and the theorem is proven. □

## 5. Convergence of the Finite Dimensional Approximations

We want to establish the convergence of the finite dimensional maximum likelihood estimators to the maximum likelihood estimator corresponding to the infinite dimensional model. As mentioned earlier, we cannot actually compute ${\overset{ˆ}{P}}_{n,m}$ in (3.6) and consequently we approximate it by ${\overset{ˆ}{P}}_{n,m,M}^{N}$ in (3.7). Consider the following assumptions,

**A1.** For all n,m, and N, the map $P\mapsto {ℒ}_{n,m}^{N}(P;\overset{ˆ}{𝓨})$ is a continuous map.

**A2.** For any $P_{M},\;P\in 𝒫(Q)$, M=1,
2,…, such that $\rho \left(P_{M},P\right)\to 0$, we have ${ℒ}_{i}^{N}\left(P_{M};{\overset{ˆ}{𝓨}}_{i}\right)\to {ℒ}_{i}\left(P;{\overset{ˆ}{𝓨}}_{i}\right)$ as N,M→∞ for i=1,…,m.

**A3.** For all P∈𝒫(Q) and $i=1,\ldots ,m,{ℒ}_{i}^{N}\left(P_{M};{\overset{ˆ}{𝓨}}_{i}\right)$ and ${ℒ}_{i}\left(P;{\overset{ˆ}{𝓨}}_{i}\right)$ are uniformly bounded.

**Theorem 5.1.** *Under assumptions* **A1–A3**, *there exists maximizers* ${\overset{ˆ}{P}}_{n,m,M}^{N}$ *given by* equation (3.7). *In addition, there exists a subsequence of* ${\overset{ˆ}{P}}_{n,m,M}^{N}$ *that converges to* ${\overset{ˆ}{P}}_{n,m}$ *given by* equation (3.6) *as* M,N→∞.

*Proof*. For all n,m, and N, by continuity of the map $P\mapsto {ℒ}_{n,m}^{N}(P;\overset{ˆ}{𝓨})$, per assumption **A1**, and compactness of (𝒫(Q),ρ), we can conclude that ${\overset{ˆ}{P}}_{n,m,M}^{N}$ exists.

In [21], it is shown that ${\tilde{𝒫}}_{d}(Q)$ given by equation (2.1) is a dense subset of 𝒫(Q). Thus, for M=1,
2,…, construct a sequence of probability measures $P_{M}\in {𝒫}_{M}(Q)\subset {\tilde{𝒫}}_{d}(Q)\subset 𝒫(Q)$, such that $\rho \left(P_{M},P\right)\to 0$ in 𝒫(Q). Then by assumptions **A2** and **A3**, we have

$$\left|{ℒ}_{n,m}^{N}\left(P_{M};\hat{𝓨}\right)-{ℒ}_{n,m}\left(P;\hat{𝓨}\right)\right|=\left|\overset{m}{\underset{i=1}{\prod }}{ℒ}_{i}^{N}\left(P_{M};{\hat{𝓨}}_{i}\right)-\overset{m}{\underset{i=1}{\prod }}{ℒ}_{i}\left(P;{\hat{𝓨}}_{i}\right)\right|\to 0.$$

Consequently, ${ℒ}_{n,m}^{N}\left(P_{M};\overset{ˆ}{𝓨}\right)\to {ℒ}_{n,m}(P;\overset{ˆ}{𝓨})$ as N,M→∞.

In addition, by definition, for each n,m, and N, and for all $P_{M}\in {𝒫}_{M}(Q)$, we have

(5.1) $${ℒ}_{n,m}^{N}\left({\hat{P}}_{n,m,M}^{N};\hat{𝓨}\right)\leq {ℒ}_{n,m}^{N}\left(P_{M};\hat{𝓨}\right).$$

In addition, by compactness of 𝒫(Q), there exists a subsequence of ${\overset{ˆ}{P}}_{n,m,M}^{N}$ that converges to ${\overset{ˆ}{P}}_{n,m}$ as M,N→∞. Thus, by taking the limit in (5.1) as M,N→∞, for all P∈𝒫(Q), we find that

$${ℒ}_{n,m}\left({\hat{P}}_{n,m};\hat{𝓨}\right)\leq {ℒ}_{n,m}\left(P;\hat{𝓨}\right);$$

thus, ${\overset{ˆ}{P}}_{n,m}=\text{arg}\;\underset{P\in 𝒫(Q)}{\text{max}}\;{ℒ}_{n,m}(P;\overset{ˆ}{𝓨})$ as given in equation (3.6). □

In practice, to achieve a desired level of accuracy, M and N are fixed sufficiently large. We choose a sufficiently large value for N, how large that needs to be, of course, depends on the particular numerical discretization scheme chosen. The most common choice would be using a Galerkin-based method to the approximate $y_{k,i}$ by $y_{k,i}^{N}$, where

$$y_{k,i}^{N}=h_{k}\left(x_{k,i}^{N}\left(q_{i}\right),\phi_{0,i}^{N},u_{k,i};q_{i}\right),$$

which denotes the discretization of the output of the model.

We also choose a sufficiently large value for M, the number of nodes, ${\left\{q_{j}\right\}}_{j=1}^{M}$. Therefore, the optimization problem is reduced to a standard constrained estimation problem over Euclidean M-space, in which we determine the values of the weights $p_{j}$ at each node $q_{j}$ with the constraints that they all be non-negative and sum to one. By equation (3.7). It follows that

$${\hat{P}}_{n,m,M}^{N}=\text{arg}\underset{P\in {𝒫}_{M}(Q)}{\text{max}}{ℒ}_{n,m}^{N}(P;\hat{𝓨})\;=\text{arg}\underset{P\in {𝒫}_{M}(Q)}{\text{max}}\prod_{i=1}^{m}\int_{Q}\prod_{k=1}^{n_{i}}\varphi \left({\hat{𝒴}}_{k,i}-y_{k,i}^{N}\left(q_{i}\right)\right)dP\left(q_{i}\right)\;=\text{arg}\underset{P\in {𝒫}_{M}(Q)}{\text{max}}\prod_{i=1}^{m}\int_{Q}\prod_{k=1}^{n_{i}}\varphi \left({\hat{𝒴}}_{k,i}-h_{k}\left(x_{k,i}^{N}\left(q_{i}\right),\phi_{0,i}^{N},u_{k,i};q_{i}\right)\right)dP\left(q_{i}\right)\;=\underset{\tilde{p}\in \tilde{{\mathbb{R}}^{M}}}{\text{arg}\;\text{max}}\prod_{i=1}^{m}\sum_{j=1}^{M}\prod_{k=1}^{n_{i}}\varphi \left({\hat{𝒴}}_{k,i}-h_{k}\left(x_{k,i}^{N},\phi_{0,i}^{N},u_{k,i};q_{j}\right)\right)p_{j}\;=\text{arg}\underset{\tilde{p}\in \tilde{{\mathbb{R}}^{M}}}{\text{max}}\prod_{i=1}^{m}\sum_{j=1}^{M}p_{j}\prod_{k=1}^{n_{i}}\varphi \left({\hat{𝒴}}_{k,i}-h_{k}\left(x_{k,i}^{N},\phi_{0,i}^{N},u_{k,i};q_{j}\right)\right)p_{j},$$

where $\tilde{p}=\left(p_{1},\ldots ,p_{M}\right)\in \tilde{R^{M}}=\left\{\tilde{p}|p_{j}\in R^{+},\sum_{j=1}^{M}\;p_{j}=1\right\}$.

We note that computing ${\overset{ˆ}{P}}_{n,m,M}^{N}$ involves high order products of very small numbers which not unexpectedly can cause numerical underflow. In order to mitigate this, we maximize the log-likelihood function instead and rewrite it in a form that lends itself to the use of the MATLAB optimization routine *logsumexp* as follows

(5.2) $${\hat{P}}_{n,m,M}^{N}=\underset{\tilde{p}\in \tilde{{\mathbb{R}}^{M}}}{\text{arg}\;\text{max}\;\text{log}}\left(\prod_{i=1}^{m}\sum_{j=1}^{M}p_{j}\prod_{k=1}^{n_{i}}\varphi \left({\hat{𝒴}}_{k,i}-h_{k}\left(x_{k,i}^{N},\phi_{0,i}^{N},u_{k,i};q_{j}\right)\right)\right)\;=\text{arg}\underset{\tilde{p}\in \tilde{{\mathbb{R}}^{M}}}{\text{max}}\sum_{i=1}^{m}\text{log}\left(\sum_{j=1}^{M}p_{j}\prod_{k=1}^{n_{i}}\varphi \left({\hat{𝒴}}_{k,i}-h_{k}\left(x_{k,i}^{N},\phi_{0,i}^{N},u_{k,i};q_{j}\right)\right)\right)\;=\text{arg}\underset{\tilde{p}\in \tilde{{\mathbb{R}}^{M}}}{\text{max}}\sum_{i=1}^{m}\text{log}\left(\sum_{j=1}^{M}\text{exp}\left(\text{log}\left(p_{j}\prod_{k=1}^{n_{i}}\varphi \left({\hat{𝒴}}_{k,i}-h_{k}\left(x_{k,i}^{N},\phi_{0,i}^{N},u_{k,i};q_{j}\right)\right)\right)\right)\right)\;=\text{arg}\underset{\tilde{p}\in \tilde{{\mathbb{R}}^{M}}}{\text{max}}\sum_{i=1}^{m}\text{log}\left(\sum_{j=1}^{M}\text{exp}\left(\text{log}\left(p_{j}\right)+\sum_{k=1}^{n_{i}}\text{log}\left(\varphi \left({\hat{𝒴}}_{k,i}-h_{k}\left(x_{k,i}^{N},\phi_{0,i}^{N},u_{k,i};q_{j}\right)\right)\right)\right)\right).$$

## 6. Abstract Parabolic Systems

In order to apply our estimation theory to equations (1.1)–(1.5), our model for the transdermal transport of ethanol given in Section 1, we reformulate it as an abstract parabolic system. We briefly describe what an abstract parabolic system is, its properties, and its finite dimensional approximation, and then we show how assumptions **A1–A3** are satisfied for such a system.

Let H and V be Hilbert spaces with V densely and continuously embedded in H. Pivoting on H, it follows that H is therefore densely and continuously embedded in the dual of $V,\;V^{*}$. This is known as a Gelfand triple and is generally written as $V↪H↪V^{*}$ [38]. Then an abstract parabolic system is a dynamical system of the following form

(6.1) $$<\dot{x},\psi {>}_{V^{*},V}+a\left(q;x,\psi \right)=<B\left(q\right)u,\psi {>}_{V^{*},V},\;\psi \in V,\;x\left(0\right)=x_{0},\;y\left(t\right)=C\left(q\right)x\left(t\right),$$

where $<\cdot ,\cdot {>}_{V^{*},V}$ denotes the duality pairing between $V^{*}$ and V,Q is as defined in Section 1, and for each q∈Q,a(q;.,.):V×V→C is a sesquilinear form satisfying the following three assumptions,

**B1. (Boundedness)** There exists a constant $\alpha_{0}$ such that for all $\psi_{1},\;\psi_{2}\in V$, we have

$$\left|a\left(q;\psi_{1},\psi_{2}\right)\right|\leq \alpha_{0}{\left\|\psi_{1}\right\|}_{V}{\left\|\psi_{2}\right\|}_{V}.$$

**B2. (Coercivity)** There exists $\lambda_{0}\in R$ and $\mu_{0}>0$ such that for all ψ∈V, we have

$$a\left(q;\psi ,\psi \right)+\lambda_{0}|\psi {|}_{H}^{2}\geq \mu_{0}\|\psi {\|}_{V}^{2}.$$

**B3. (Continuity)** For all $\psi_{1},\;\psi_{2}\in V$ and $q,\;\tilde{q}\in Q$, we have

$$\left|a\left(q,\psi_{1},\psi_{2}\right)-a\left(\tilde{q},\psi_{1},\psi_{2}\right)\right|\leq d\left(q,\tilde{q}\right){\left\|\psi_{1}\right\|}_{V}{\left\|\psi_{2}\right\|}_{V}.$$

In these assumptions, $\|\cdot {\|}_{V}$ and $|\cdot {|}_{H}$ denotes the norm on the spaces V and H, respectively. Further, in (6.1), $B(q):R^{V}\to V^{*}$, and C(q):V→R are bounded linear operators with initial conditions $x_{0}\in H$, input $u\in L^{2}\left([0,T],R^{v}\right)$, and output $y\in L^{2}([0,T],R)$.

It can be shown that the system in (6.1) has a unique solution in

$$\left\{\psi |\psi \in L^{2}\left(\left[0,T\right],V\right),\dot{\psi }\in L^{2}\left(\left[0,T\right],V^{*}\right)\right\}\subset C\left(\left[0,T\right],H\right)$$

using standard variational arguments (such as in [39]). However, we use a linear semigroup approach to convert the system in (6.1) into a discrete-time state space model and then use arguments from linear semigroup theory [6, 40] to argue convergence of finite dimensional Galerkin-based approximations and conclude that assumptions **A1–A3** are satisfied.

Assumptions **B1** and **B2** yield that the form a(q;.,.) defines a bounded linear operator $A(q):V\to V^{*}$ given by

$$<A\left(q\right)\psi_{1},\psi_{2}{>}_{V^{*},V}=-a\left(q;\psi_{1},\psi_{2}\right),$$

for $\psi_{1},\;\psi_{2}\in V$. If we restrict the operator A(q) to the subspace

Dom(A(q))={ψ∈V∣A(q)ψ∈H},

it becomes the infinitesimal generator of a holomorphic or analytic semigroup, $\left\{e^{A(q)t}|t\geq 0\right\}$, of bounded linear operators on H. The operator A(q) is referred to as being regularly dissipative [5,6,38]. Moreover, this semigroup can also be extended and restricted to be a holomorphic semigroup on $V^{*}$ and V, respectively [5, 38].

The system in (6.1) can now be written in state space form with time invariant operators A(q), **B(q)**, and C(q), as

(6.2) $$\dot{x}\left(t\right)=A\left(q\right)x\left(t\right)+B\left(q\right)u\left(t\right)\;x\left(0\right)=x_{0},\;y\left(t\right)=C\left(q\right)x\left(t\right).$$

The operator form of the variation of constants formula then yields what is known as a mild solution of (6.2) given by

(6.3) $$x\left(t;q\right)=e^{A\left(q\right)t}x_{0}+\int_{0}^{t}e^{A\left(q\right)\left(t-s\right)}B\left(q\right)u\left(s\right)ds,\;t\geq 0,\;y\left(t;q\right)=C\left(q\right)x\left(t;q\right).$$

To obtain the corresponding discrete or sampled time form of the system given in (6.2) or (6.3), let τ>0 be the length of the sampling interval, and consider strictly zero-order hold inputs of the form $u(t)=u_{k-1},\;t\in [(k-1)\tau ,k\tau )$, k=1,
2,…. Then, let $x_{k}=x(k\tau )$ and $y_{k}=y(k\tau )$, k=1,
2,…. By applying (6.3) on each sub-interval [(k-1)τ,kτ], k=1,
2,…, we obtain the discrete-time dynamical system given by

(6.4) $$x_{k}=\hat{A}\left(q\right)x_{k-1}+\hat{B}\left(q\right)u_{k-1},\;k=1,2,\ldots ,$$

(6.5) $$y_{k}=\hat{C}\left(q\right)x_{k},\;k=1,2,\ldots ,$$

where $x_{0}\in V,\;\overset{ˆ}{A}(q)=e^{A(q)\tau },\;\overset{ˆ}{B}(q)=\int_{0}^{\tau }\;e^{A(q)s}B(q)ds$, and $\overset{ˆ}{C}(q)=C(q)$.

Using a standard Galerkin approach [41], we can approximate the discrete-time system given in (6.4)–(6.5) by a sequence of approximating finite dimensional discrete-time systems in a sequence of finite dimensional subspaces, $V^{N}$, of V. In order to argue convergence, we will require the following additional assumption concerning the subspaces $V^{N}$,

**C1. (Approximation)** For every x∈V, there exists $x^{N}\in V^{N}$ such that ${\left\|x-x^{N}\right\|}_{V}\to 0$ as N→∞.

We consider the sequence of approximating finite dimensional discrete-time systems by

$$x_{k}^{N}={\hat{A}}^{N}\left(q\right)x_{k-1}^{N}+{\hat{B}}^{N}\left(q\right)u_{k-1},k=1,2,\ldots ,\;y_{k}^{N}={\hat{C}}^{N}\left(q\right)x_{k}^{N},k=1,2,\ldots ,$$

where ${\overset{ˆ}{A}}^{N}(q)=e^{A^{N}(q)\tau },\;{\overset{ˆ}{B}}^{N}(q)=\int_{0}^{\tau }\;e^{A^{N}(q)s}B^{N}(q)ds$, and ${\overset{ˆ}{C}}^{N}(q)x_{k}=\overset{ˆ}{C}(q)$, where for each $q\in Q,\;A^{N}(q)$ is the linear operator on $V^{N}$ obtained by restricting the form a(q;.,.) to $V^{N}\times V^{N}$, i.e. for $\psi_{1}^{N},\;\psi_{2}^{N}\in V^{N}$,

$$<A^{N}\left(q\right)\psi_{1}^{N},\psi_{2}^{N}{>}_{V^{*},V}=-a\left(q;\psi_{1}^{N},\psi_{2}^{N}\right).$$

And also, $B^{N}(q)=\pi^{N}B(q)$, where in this definition, $\pi^{N}$ is the natural extension of the orthogonal projection operator $\pi^{N}:H\to V^{N}$ to $V^{*}$ from its dense subspace H. We also set $x_{0}^{N}=\pi^{N}x_{0}\in V^{N}$.

Under the assumptions **B1–B3** and **C1** using the Trotter-Kato approximation theorem from the theory of linear semigroups of operators [40, 42], we were able to conclude that ${\text{lim}}_{N\to \infty }\;{\left\|x_{k}^{N}-x_{k}\right\|}_{V}=0$ and ${\text{lim}}_{N\to \infty }\;\left|y_{k}^{N}-y_{k}\right|=0$ for each $x_{0}\in V$, and uniformly in q for q∈Q and k∈{1,
2,…,K}, for any fixed $K\in N^{+}$.

We can now use the results described in the previous paragraphs to show that an abstract parabolic system satisfies assumptions **A1–A3** given in Section 5. To show that the assumption **A1** is satisfied, we need to show that for all n,m, and N, the map $P\mapsto {ℒ}_{n,m}^{N}(P;\overset{ˆ}{𝓨})$ is a continuous map. It suffices to show that for any fixed n,m, and N, and for any sequence of probability measures $P_{M}$, such that $\rho \left(P_{M},P\right)\to 0$ in 𝒫(Q), we have ${ℒ}_{n,m}^{N}\left(P_{M};\overset{ˆ}{𝓨}\right)\to {ℒ}_{n,m}^{N}(P;\overset{ˆ}{𝓨})$ as M→∞. Towards this end, we see that

$$\left|{ℒ}_{n,m}^{N}\left(P_{M};\hat{𝓨}\right)-{ℒ}_{n,m}^{N}\left(P;\hat{𝓨}\right)\right|=\left|\overset{m}{\underset{i=1}{\prod }}{ℒ}_{i}^{N}\left(P_{M};{\hat{𝓨}}_{i}\right)-\overset{m}{\underset{i=1}{\prod }}{ℒ}_{i}^{N}\left(P;{\hat{𝓨}}_{i}\right)\right|\;=|\overset{m}{\underset{i=1}{\prod }}\int_{Q}\overset{n_{i}}{\underset{k=1}{\prod }}\varphi \left({\hat{𝒴}}_{k,i}-y_{k,i}^{N}\left(q_{i}\right)\right)dP_{M}\left(q_{i}\right)-\overset{m}{\underset{i=1}{\prod }}\int_{Q}\overset{n_{i}}{\underset{k=1}{\prod }}\varphi \left({\hat{𝒴}}_{k,i}-y_{k,i}^{N}\left(q_{i}\right)\right)dP\left(q_{i}\right)|\;\to 0,$$

by definition of the Prohorov metric. It follows that the assumption **A1** is satisfied.

Next, we show that the assumption **A2** is satisfied. We have that ${\text{lim}}_{N\to \infty }\;{\left\|x_{k,i}^{N}-x_{k,i}\right\|}_{V}=0$ and ${\text{lim}}_{N\to \infty }\;\left|y_{k,i}^{N}-y_{k,i}\right|=0$ for each $x_{0,i}\in V$, uniformly in $q_{i}$ for $q_{i}\in Q,\;k=1,\ldots ,n_{i},\;i=1,\ldots ,m$. We want to show that for any sequence of probability measures $P_{M}$, such that $\rho \left(P_{M},P\right)\to 0$ in 𝒫(Q), and for i=1,…,m, as N,M→∞, we have ${ℒ}_{i}^{N}\left(P_{M};{\overset{ˆ}{𝓨}}_{i}\right)\to {ℒ}_{i}\left(P;{\overset{ˆ}{𝓨}}_{i}\right)$.

Recall that φ is assumed to be continuous. Let ε>0, and choose $N_{0}$ such that for $N\geq N_{0}$, and for every $M,\left|\int_{Q}\;\left(\prod_{k=1}^{n_{i}}\;\varphi \left({\overset{ˆ}{𝒴}}_{k,i}-y_{k,i}^{N}\left(q_{i}\right)\right)-\prod_{k=1}^{n_{i}}\;\varphi \left({\overset{ˆ}{𝒴}}_{k,i}-y_{k,i}\left(q_{i}\right)\right)\right)dP_{M}\left(q_{i}\right)\right|<\varepsilon /2$. Then, we have

$$\left|{ℒ}_{i}^{N}\left(P_{M};{\hat{𝓨}}_{i}\right)-{ℒ}_{i}\left(P;{\hat{𝓨}}_{i}\right)\right|\;=\left|\int_{Q}\overset{n_{i}}{\underset{k=1}{\prod }}\varphi \left({\hat{𝒴}}_{k,i}-y_{k,i}^{N}\left(q_{i}\right)\right)dP_{M}\left(q_{i}\right)-\int_{Q}\overset{n_{i}}{\underset{k=1}{\prod }}\varphi \left({\hat{𝒴}}_{k,i}-y_{k,i}\left(q_{i}\right)\right)dP\left(q_{i}\right)\right|\;\leq \left|\int_{Q}\left(\overset{n_{i}}{\underset{k=1}{\prod }}\varphi \left({\hat{𝒴}}_{k,i}-y_{k,i}^{N}\left(q_{i}\right)\right)-\overset{n_{i}}{\underset{k=1}{\prod }}\varphi \left({\hat{𝒴}}_{k,i}-y_{k,i}\left(q_{i}\right)\right)\right)dP_{M}\left(q_{i}\right)\right|\;+\left|\int_{Q}\overset{n_{i}}{\underset{k=1}{\prod }}\varphi \left({\hat{𝒴}}_{k,i}-y_{k,i}\left(q_{i}\right)\right)dP_{M}\left(q_{i}\right)-\int_{Q}\overset{n_{i}}{\underset{k=1}{\prod }}\varphi \left({\hat{𝒴}}_{k,i}-y_{k,i}\left(q_{i}\right)\right)dP\left(q_{i}\right)\right|\;<\frac{\varepsilon }{2}+\frac{\varepsilon }{2}=\varepsilon ,$$

where the second term is less than ε/2 by definition of the Prohorov metric. Consequently, the assumption **A2** is satisfied.

Finally, we want to show that the assumption **A3** is satisfied. We want to show that for all P∈𝒫(Q) and for $i=1,\ldots ,m,{ℒ}_{i}^{N}\left(P_{M};{\overset{ˆ}{𝓨}}_{i}\right)$ and ${ℒ}_{i}\left(P;{\overset{ˆ}{𝓨}}_{i}\right)$ are uniformly bounded. Recall that the parameter space Q is compact. Thus, for $q_{i}\in Q$, and for each $N,\;y_{k,i}^{N}\left(q_{i}\right)$ are uniformly bounded. Similarly, $y_{k,i}\left(q_{i}\right)$ are also uniformly bounded and we also have that $\left|y_{k,i}^{N}\left(q_{i}\right)-y_{k,i}\left(q_{i}\right)\right|\to 0$ uniformly in $q_{i}$ for $q_{i}\in Q$. Therefore, we can conclude that the assumption **A3** is satisfied.

## 7. Application to the Transdermal Transport of Alcohol

To apply the results established in Section 6 to the system (1.1)–(1.5) in Section 1, the system must first be written in weak form. Then, the parameter space Q, the Hilbert spaces H and V, the sesquilinear form a(q;.,.), and the operators B(q) and C(q) must all be identified. Also, the approximating subspaces, $V^{N}$, must be chosen, and finally assumptions **B1**-**B3** and **C1** must all be shown to be satisfied.

The parameter space Q is assumed to be a compact subset of $R^{+}\times R^{+}$ with any p-metric denoted by $d_{Q}$. Let $x=\left(x_{1},\ldots ,x_{n}\right)\in R^{n}$ and $y=\left(y_{1},\ldots ,y_{n}\right)\in R^{n}$, then the p-metric is defined by

$$d_{p}\left(x,y\right)={\left(\overset{n}{\underset{i=1}{\sum }}{\left|x_{i}-y_{i}\right|}^{p}\right)}^{1/p}$$

for any p∈[1,∞). For p=∞, the p-metric is defined by

$$d_{p}\left(x,y\right)=\underset{i=1,\ldots ,n}{\text{max}}\left|x_{i}-y_{i}\right|.$$

Let $H=L^{2}(0, 1)$ and $V=H^{1}(0, 1)$ with their standard inner products and norms. It follows that $V^{*}=H^{-1}(0, 1)$, and the three spaces H,V, and $V^{*}$ form a Gelfand triple. To rewrite the system (1.1)–(1.5) in weak form, we multiply by a test function ψ∈V and integrate by parts to obtain

$$<\dot{x}\left(t\right),\psi {>}_{V^{*},V}+\int_{0}^{1}q_{1}\frac{\partial x}{\partial \eta }\left(t,\eta \right)\psi^{\prime }\left(\eta \right)d\eta +x\left(t,0\right)\psi \left(t,0\right)=q_{2}u\left(t\right)\psi \left(1\right),$$

where $<\cdot ,\cdot {>}_{V^{*},V}$ denotes the duality pairing between $V^{*}$ and V. Then for q∈Q,u∈R, and $\tilde{\psi },\;\psi \in V$, we set

$$a\left(q;\tilde{\psi },\psi \right)=\int_{0}^{1}q_{1}\tilde{\psi }\prime \left(\eta \right)\psi^{\prime }\left(\eta \right)d\eta +\tilde{\psi }\left(0\right)\psi \left(0\right),\;<B\left(q\right)u,\psi {>}_{V^{*},V}=q_{2}u\psi \left(1\right),\;C\left(q\right)\psi =C\psi =\psi \left(0\right).$$

We can establish that assumptions **B1–B3** are satisfied using arguments involving the Sobolev Embedding Theorem (see [43]). Also, the operators B(q) and C(q) are continuous in the uniform operator topology with respect to q∈Q. It follows from Section 6 that

$$g_{k-1}\left(x_{k-1,i}\left(q_{i}\right),u_{k-1,i};q_{i}\right)=\hat{A}\left(q_{i}\right)x_{k-1,i}\left(q_{i}\right)+\hat{B}\left(q_{i}\right)u_{k-1,i},\;k=1,2,\ldots ,n_{i},i=1,\ldots ,m,\;h_{k}\left(x_{k,i}\left(q_{i}\right),\phi_{0,i},u_{k,i};q_{i}\right)=\hat{C}\left(q_{i}\right)x_{k,i}\left(q_{i}\right),\;k=1,\ldots ,n_{i},i=1,\ldots ,m,$$

where $\overset{ˆ}{A}(q)=e^{A(q)\tau },\;\overset{ˆ}{B}(q)=\int_{0}^{\tau }\;e^{A(q)s}B(q)ds$, and $\overset{ˆ}{C}(q)=C(q)$ with τ>0 the length of the sampling interval.

Let $V^{N},\;N=1, 2,\ldots$, be the span of the standard linear splines defined with respect to the uniform mesh {0,
1/N,2/N,…,(N-1)/N,1} on [0, 1]. Then, assumption **C1** is satisfied by standard arguments for spline functions (see, for example, [44]). If for each i=1,
2,…,m, we define $x_{k,i}^{N}$ and $y_{k,i}^{N}$ as in (6.4)–(6.5), then by the arguments at the end of Section 6, we conclude that assumptions **A1–A3** are satisfied.

In the following two subsections, 7.1 and 7.2, we present the application of our scheme to the transdermal transport of alcohol in two examples, one involving simulated data, and the other using actual human subject data collected in the Luczak laboratory at USC. For the simulated data, we want to show the convergence of the estimated distribution of the parameter vector $q=\left(q_{1},q_{2}\right)$ to the “true” distribution as the number of drinking episodes increases, as the number of nodes increases, and as the level of discretization in the finite dimensional approximations increases. And, for the actual data, we apply the leave-one-out cross-validation (LOOCV) method by estimating the distribution of the parameter vector q using the training set, and then estimating the TAC output using the estimated distribution and the BrAC input of the test set.

### 7.1. Example 1: Estimation Based on Simulation Data

In this example, we estimate the distribution of the parameter vector $q=\left(q_{1},q_{2}\right)$ in the system (1.1)–(1.5) by first simulating TAC data in MATLAB with the assumption that the two parameters $q_{1}$ and $q_{2}$ are i.i.d. with a Beta distribution, $q_{1},\;q_{2}\sim \text{Beta}(2, 5)$. Thus, their joint cumulative distribution function (cdf) is the product of their marginal Beta(2,
5) cdfs.

From equation (3.1), we have

$$Y_{k,i}=y_{k,i}\left(q_{i}\right)+e_{k,i},\;k=1,\ldots ,n_{i},\;i=1,\ldots ,m,$$

where m is the number of drinking episodes, and $y_{k,i}\left(q_{i}\right)$ is the observed TAC for the $i^{\text{th}}$ drinking episode at time step k. We let $P_{0}$ be the product of the cdfs of two independent Beta(2,5) distributions, and $e_{k,i}\sim N\left(0,10^{-6}\right),\;k=1,\ldots ,n_{i},\;i=1,\ldots ,m$. The reason for the small variance is because the noise accounts for errors in the measurements caused by factors that result in small perturbations to the measurements, such as humidity or temperature as external environmental factors, or the level of skin moisture or greasiness among different drinking episodes. It is assumed that these types of factors are normally distributed causing small variations to the measurements.

To approximate the PDE model for the TAC observations, we used the linear spline-based Galerkin approximation scheme described in Section 6 with N equally spaced sub-intervals from [0, 1] (see [15–17]). We want to compute ${\overset{ˆ}{P}}_{n,m,M}^{N}$ given by equation (5.2), where $q_{j}=\left(q_{j_{1}},q_{j_{2}}\right)$ is chosen as M uniform meshgrid coordinates on [0, 1] × [0, 1]. We make the assumption that there is no alcohol in the epidermal layer of the skin at time t=0, so we let $\phi_{0,i}^{N}=0$. The constrained optimization problem over Euclidean M-space was solved using constrained optimization routine FMINCON from the Optimization Toolbox in MATLAB applied to the negative of the log-likelihood function.

We note that before turning to a mixed effects statistical model, we had considered an output or observation that was aggregated TAC; in this case, the appropriate underlying statistical model was the naive pooled model (i.e. the data point for each drinking episode at a certain time is an observation of the mean behavior plus a random error). However, it is not difficult to show that when this observation is used, if the **q**_1_ and **q**_2_ are assumed to be independent, then their joint distribution is not identifiable. Consequently, when the nonlinear least squares-based constrained optimization problem is solved, the inherent ill-posedness of the inverse problem results in undesirable oscillations. To mitigate this behavior, we had to introduce an appropriately weighted regularization term in the performance index being minimized. One advantage of the mixed effects statistical model presented here is that regularization is not required.

In order to demonstrate the consistency of our estimator, we show that as m, the number of drinking episodes, increases, the estimated cdf of the parameter vector $q=\left(q_{1},q_{2}\right)$ approaches the “true” cdf, the product of two Beta(2,
5) cdfs. In order to simulate realistic longitudinal TAC vectors representing data that might be collected by the TAC biosensor for an individual’s drinking episode, we used BrAC data collected in the Luczak laboratory as the input to the model, and generated random samples of $q_{1}$ and $q_{2}$, i.i.d. Beta(2,
5) in MATLAB. Using the algorithm developed in the current paper, we estimated the distribution of the random parameter vector by solving the optimization problem for different cases based on the number of drinking episodes, m, and observed the convergence of the estimated distribution to the “true” distribution as m increases.

To quantify this, let D be the sum of the squared differences at each node between the estimated and the “true” distribution, the product of two Beta(2,
5) cdfs. Let $p_{j}$ and $b_{j}$ be the weights at the node $q_{j}$ of the estimated and “true” distribution, respectively. So, we have

$$D=\overset{M}{\underset{j=1}{\sum }}{\left(p_{j}-b_{j}\right)}^{2},$$

representing the error in estimating the weights at each node. This is a metric being used to quantify that the difference between the estimated distribution and the “true” distribution decreases as the number of drinking episodes increases.

We fixed the number of nodes, M, and the level of discretization, N, sufficiently large. We set M=400 and N=128. We estimated the distribution of the parameter vector for different cases based on different numbers of drinking episodes, m∈{1,
3, 7, 9, 16,
42}, and calculated D for each case. Our results are summarized in Table 1. We observed that as the number of drinking episodes, m, increases, the sum of the squared differences at each node between the estimated distribution and the “true” distribution, D, decreases.

**Figure 2.**
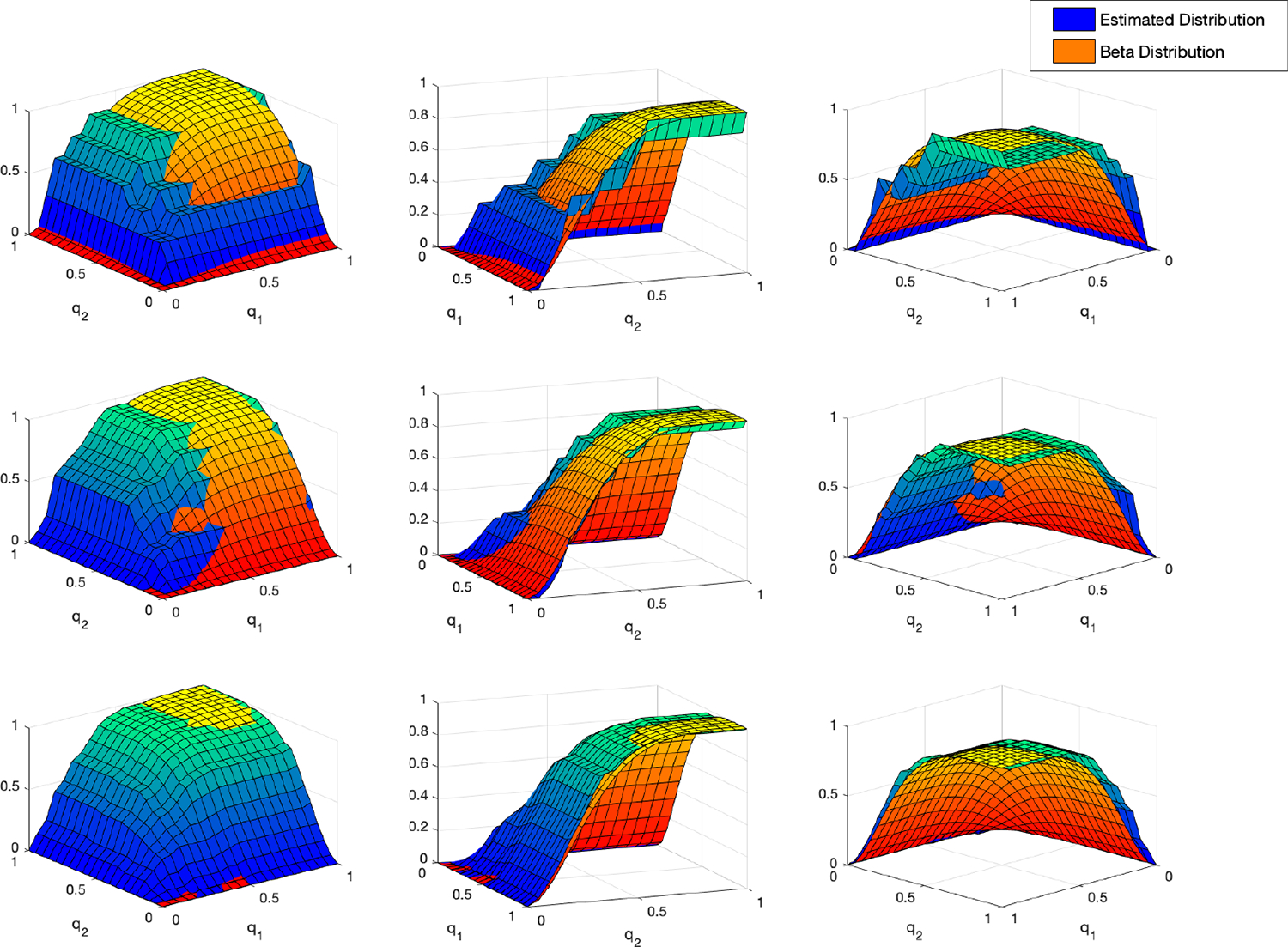
Each row of the figure contains three different views of the same plot of the estimated distribution and the “true” joint Beta(2,
5) distribution for different numbers of drinking episodes m=7,m=16, and m=42 in the top, middle, and bottom rows, respectively, for a fixed number of nodes M=400 and level of discretization N=128. We observe that as m increases from the top row to the bottom row, our estimated distribution gets “closer” to the “true” distribution, which agrees with the numerical results that are shown in Table 1.

In Figure 2, each row of the figure contains three different views of the same plot of the estimated distribution and the “true” distribution (again, the product of two Beta(2,
5) cdfs) for different numbers of drinking episodes m=7,m=16, and m=42 in the top, middle, and bottom rows, respectively, with the number of nodes set to M=400 and the level of discretization to N=128. We observe that as m increases, our estimated distribution gets “closer” to the “true” distribution, which agrees with the numerical results that are shown in Table 1.

Next, we show that as the number of nodes M and the level of discretization N increases, the normalized sum of squared differences at each node between the estimated and the “true” distribution decreases.

First, we fixed the level of discretization at N=128, and we increased the number of nodes M. Let

$${\bar{D}}_{M}=\frac{1}{M}\overset{M}{\underset{j=1}{\sum }}{\left(p_{j}-b_{j}\right)}^{2}.$$

In Table 2, we observe that for the fixed value of N=128, as the number of nodes, M, increases, the normalized sum of the squared differences at each node between the estimated distribution and the “true” distribution, ${\overset{‾}{D}}_{M}$, decreases.

Next, we fixed the number of nodes at M=400, and we increased the level of discretization N. Let

$${\bar{D}}_{N}=\frac{1}{N}\overset{M}{\underset{j=1}{\sum }}{\left(p_{j}-b_{j}\right)}^{2}.$$

In Table 3, we observe that for N=128 fixed, as the number of nodes, M, increases, the normalized sum of the squared differences at each node between the estimated distribution and the “true” distribution, ${\overset{‾}{D}}_{N}$, decreases.

The choice of the “true” distribution for $q_{1}$ and $q_{2}$ in the simulation case is the scatterplot of samples for a set of 18 drinking episodes including BrAC and TAC measurements of different individuals obtained by a deterministic approach in [22]. However, the Beta(2,
5) distribution was chosen strictly for the purpose of demonstration. When applying our algorithm to actual clinic or lab collected human subject data, a valuable feature of our developed methodology in using a nonparametric approach is that we do not need to make any assumptions about the family of feasible distributions for the parameter vector unlike the parametric approach in [15–17]. In addition, the independent and identically distributed assumption was also very simplistic given that $q_{1}$ and $q_{2}$ parameters depend on the same individual and environmental conditions at the time of measurements. In the nonparametric approach, there is no need to make any assumptions about the distribution, resulting in relaxing the restrictive assumption of the distribution being independent and identically distribution. Thus, this assumption is relaxed in the flexible nonparametric approach used in the next example.

### 7.2. Example 2: Estimation Based on Actual Human Subject Data

The two datasets used in this example were obtained by two different alcohol biosensors; SCRAM CAM^®^ and WrisTAS^™^ 7[45,46]. We fixed the number of nodes at M=400 and the level of discretization at N=128, both sufficiently large with respect to convergence as we observed in our simulation data examples. From each dataset, we chose m=9 different drinking episodes. We split the drinking episodes into a training set consisting of 8 drinking episodes, and a testing set consisting of 1 drinking episode. This way, we could apply the leave-one-out cross-validation (LOOCV) method. We repeated this partitioning process 9 times, each time leaving out a different drinking episode. Using the training set, we first estimated the distribution of the parameter vector $q=\left(q_{1},q_{2}\right)$. Next, we sampled 100 parameter vectors $q=\left(q_{1},q_{2}\right)$ from the estimated distribution, and using those along with the BrAC input from the testing dataset, we simulated 100 TAC longitudinal signals. From these 100 simulated TAC signals, we estimated the “true” TAC by computing the mean at each time, and we provided what we refer to as a 95% conservative error band, or simply as a 95% error band, by taking the 2.5 and 97.5 percentiles. This approach for the error band is also used for a number of statistics associated with the TAC curve that are of particular interest to researchers and clinicians working in the area of alcohol use disorder.

**Figure 3.**
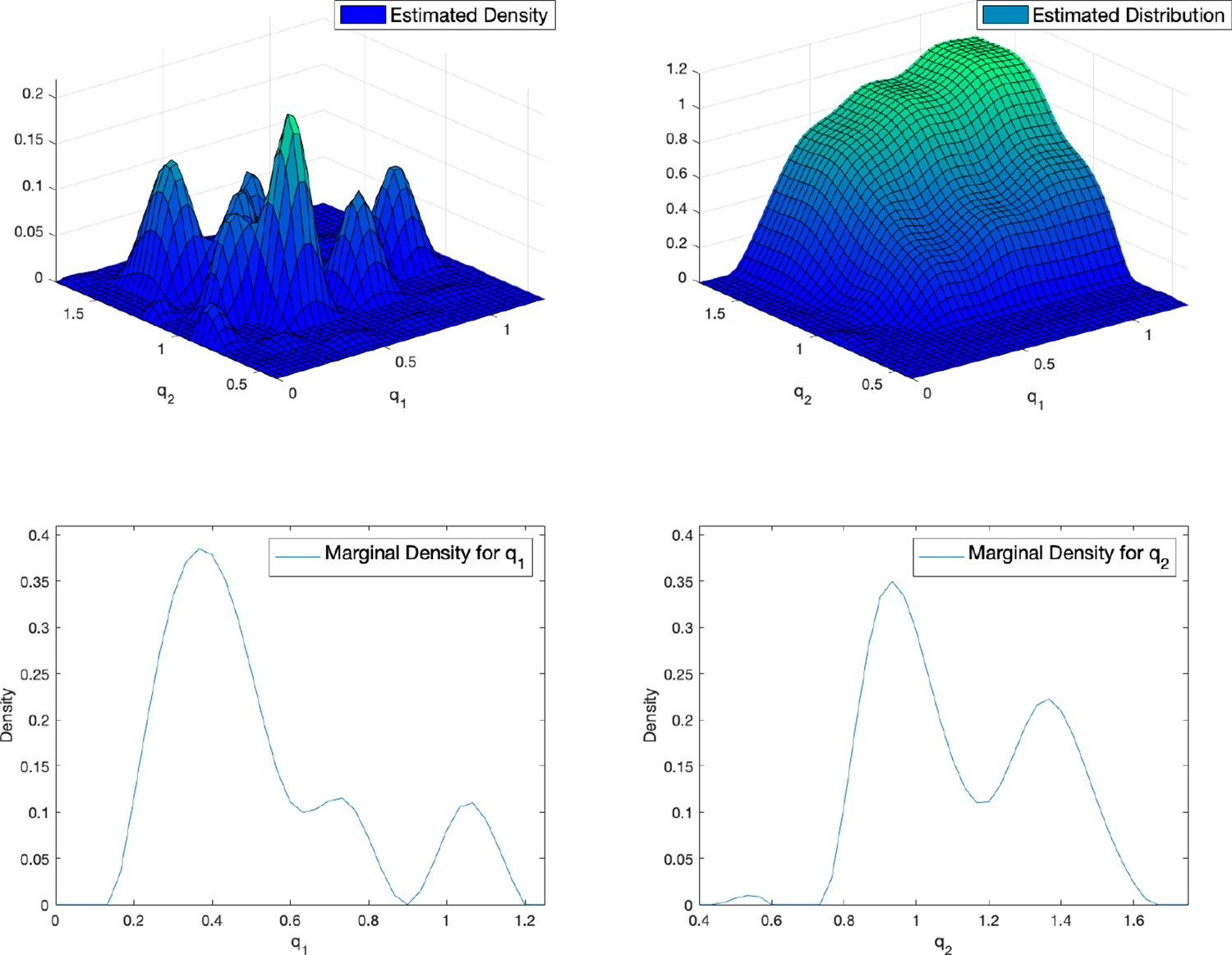
The estimated probability density function (pdf) (top left), the estimated probability distribution function (cdf) (top right), marginal density of $q_{1}$ (bottom left), and marginal density of $q_{2}$ (bottom right) obtained from m=9 drinking episodes collected using the SCRAM Alcohol Biosensor, for a fixed number of nodes M=400 and level of discretization N=128. In the estimated density plot, we can see that our numerical result for this example is in agreement with the theoretical result in Lindsay and Mallet [35, 36], which states that the maximum likelihood estimator can be found in the class of discrete distributions with at most m support points, where M≤m. In this example, since we had 9 drinking episodes, the estimated density plot displays the support points among 400 nodes for $q=\left(q_{1},q_{2}\right)$.

For the first example, we considered the dataset collected using the SCRAM alcohol biosensor. Prior to applying the leave-one-out cross-validation (LOOCV) method, in order to visualize the estimated density and distribution of $q=\left(q_{1},q_{2}\right)$, and the marginal densities of $q_{1}$ and $q_{2}$, we trained the algorithm on all 9 drinking episodes. Figure 3 illustrates the four aforementioned plots. In the estimated density plot, we can see that our numerical result for this example is in agreement with the theoretical result in Lindsay and Mallet [35, 36], which states that the maximum likelihood estimator ${\overset{ˆ}{P}}_{n,m}$ can be found in the class of discrete distributions with at most m support points, i.e. ${\overset{ˆ}{P}}_{n,m}\in {𝒫}_{M}(Q)$, where M≤m. In this example, since we had 9 drinking episodes, the estimated density plot displays the support points among 400 nodes for $q=\left(q_{1},q_{2}\right)$.

In addition, for this sample, the sample mean of $q=\left(q_{1},q_{2}\right)$ is calculated to be

$$\bar{q}=\left(0.6003,1.2452\right),$$

the sample covariance matrix is calculated to be

$$S_{q}=\left(\begin{array}{cc} 0.0706 & -0.0264\\ -0.0264 & 0.0483 \end{array}\right),$$

and the sample correlation is calculated to be $\rho_{q}=-0.4519$. Based on this, we observe that for our training population consisting of 9 drinking episodes there is a moderate negative association between the parameters $q_{1}$ and $q_{2}$. Recall that the parameters $q_{1}$ and $q_{2}$ in the system (1.1)–(1.5) represent the normalized diffusivity and the normalized flux gain at the boundary between the dermal and epidermal layers, respectively. It is important to note that the negative correlation is specifically for this 9 drinking episodes, and it should not be generalized as the behavior expected in a different sample. In particular, in the next example, we have a moderate positive correlation. In addition, it is possible to argue heuristically why one would expect either negative or positive correlation. Significantly more data and testing would be required for a statistical analysis with sufficient power to determine if any conclusion regarding the correlation between the parameters could be drawn.

**Figure 4.**
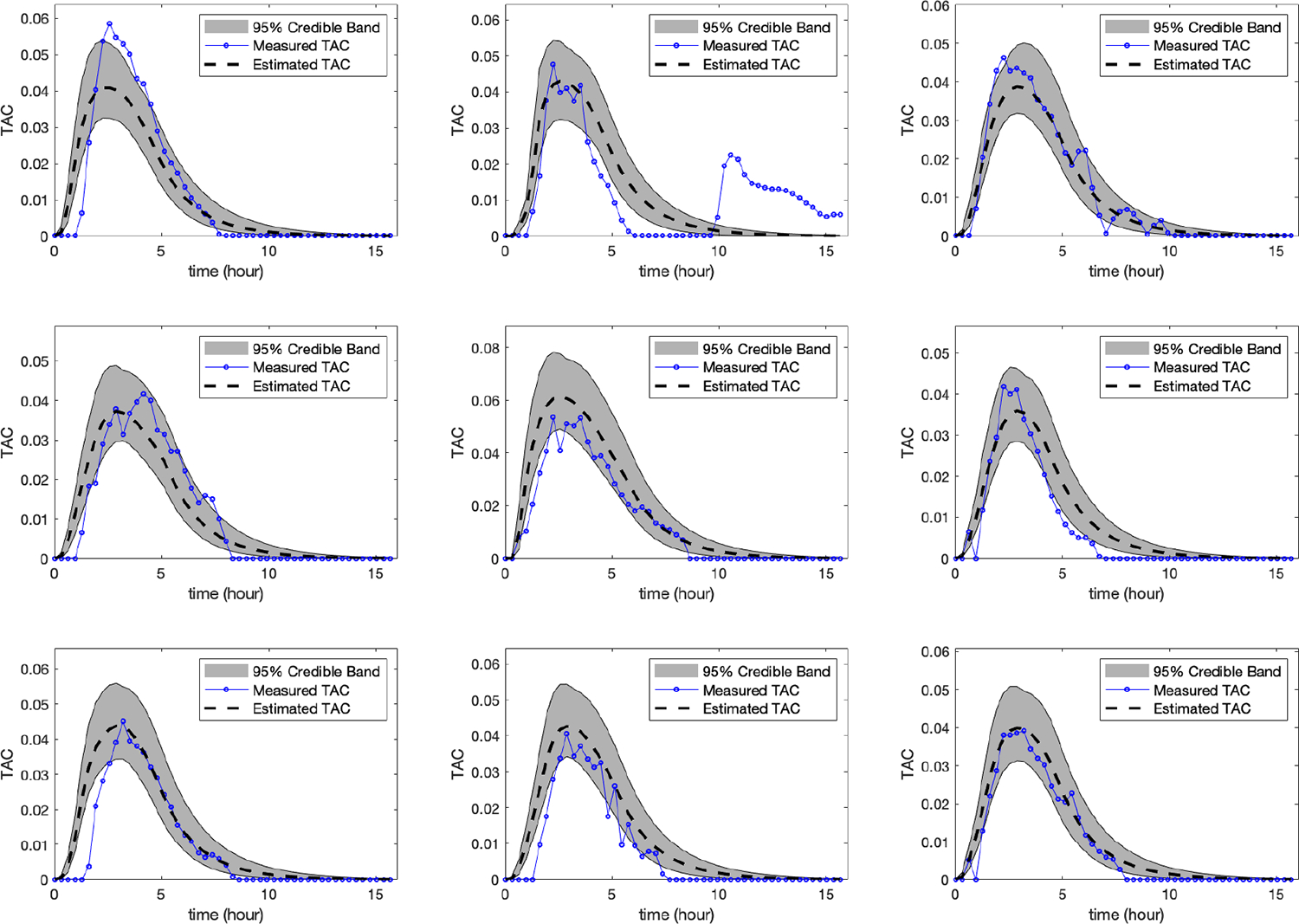
The measured TAC, the estimated TAC, and the conservative 95% error band for 9 drinking episodes from the testing set collected using the SCRAM alcohol biosensor using the LOOCV. The figure illustrates a reasonable fit. The second peak in the mid panel of the top row was due to the individual leaving the lab on a warm day which results in a burst of TAC. This error in the measurement was ignored by the model since this particular test episode was trained based on the remaining 8 drinking episodes which shows that TAC goes to zero as time increases.

We applied the leave-one-out cross-validation (LOOCV) method as explained above to the 9 drinking episodes from the SCRAM biosensor. Figure 4 shows the measured TAC (i.e. measured by the SCRAM alcohol biosensor) and the estimated TAC (i.e. obtained from our algorithm) for all the 9 drinking episodes left out in the testing set in the partitioning process, and the conservative 95% error band for a fixed number of nodes M=400 and level of discretization N=128.

Alcohol researchers and clinicians are particularly interested in a certain statistics associated with drinking episodes: the maximum or peak value of the TAC curve, the time at which the peak value of the TAC is attained, and the area under the TAC curve. The area under the curve (AUC) is a quantifying measure of exposure to the alcohol that integrates the transdermal alcohol concentration across time. Tables 4–6 display these statistics along with the measured (or actual) value obtained by the SCRAM alcohol biosensor as well as the conservative 95% error band for the 9 drinking episodes from the testing set. From these tables, we can observe that the 95% error bands do a reasonably good job of capturing the actual values of these statistics specially for the value of the peak TAC and the area under the curve displayed in Table 4 and 6. In Figure 4, we can see that there are minor fluctuations in the measured TAC curve. If we smooth the measured TAC curve, we will obtain better results in lowering the error between the time at which the peak value of the TAC is attained using the smoothed TAC curve and the estimated peak time compared to the results obtained in Table 5. The smoothed version results are included in Table 10 in the Supplementary Section 9.

**Figure 5.**
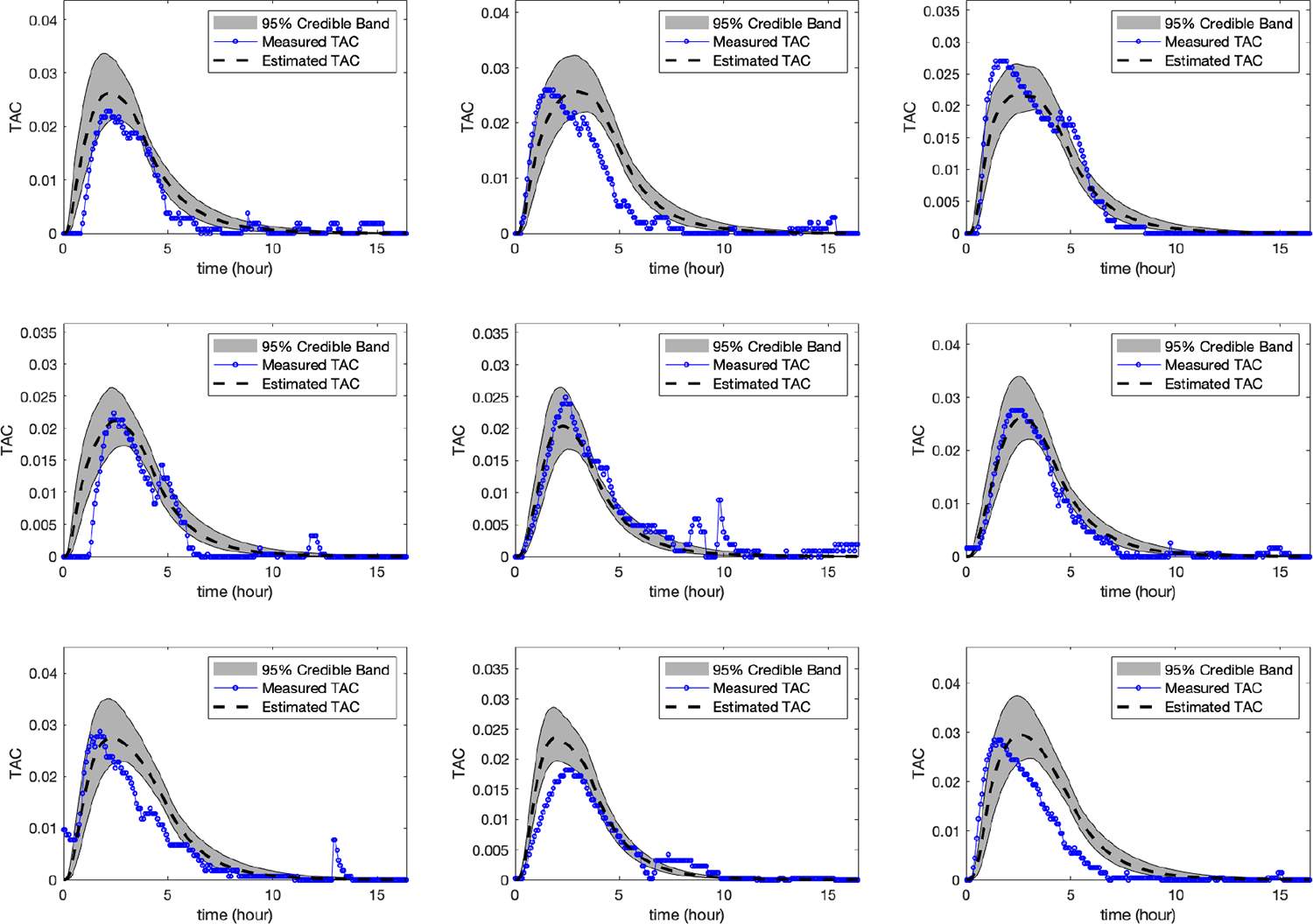
The measured TAC, the estimated TAC, and the conservative 95% error band for 9 drinking episodes from the testing set collected using the WrisTAS*^™^*7 alcohol biosensor using the LOOCV. The figure illustrates a reasonable fit.

For the second example, we applied the leave-one-out cross-validation (LOOCV) method, as explained before, to the 9 drinking episodes from the WrisTAS*^™^*7 alcohol biosensor. Figure 5 shows the measured TAC (i.e. measured by the WrisTAS*^™^*7 alcohol biosensor) and the estimated TAC (i.e. obtained from our algorithm) for all the 9 drinking episodes left out in the testing set in the partitioning process, and the conservative 95% error band for a fixed number of nodes M=400 and level of discretization N=128. We can observe that we obtained similar results as the previous example which illustrates the consistency of the developed algorithm across different alcohol biosensors.

In fact, Figure 5 shows a better fit than Figure 4, and this might be due to the fact that the WrisTAS*^™^*7 biosensor collected data more frequently (i.e. in a smaller time intervals) which resulted in more data points for the training purposes of the model. Another reason may be the difference between the accuracy of the biosensors which cannot be tested using the dataset used in this paper due to several confounding factors since the two collections of 9 drinking episodes in each example do not represent the same drinking episodes; that is, we do not have data collection by the two biosensors under the same conditions to eliminate the confounding factors. If the scientific question that is being addressed is the accuracy of the biosensors, then the researchers should collect data using the two biosensors simultaneously during the same drinking episode to eliminate the confounding factors.

Similar to the previous example using the SCRAM biosensor, in this example, using the WrisTAS*^™^*7 biosensor, we have computed some statistics as the means of comparison between the two datasets. The sample mean of $q=\left(q_{1},q_{2}\right)$ is calculated to be

$$\bar{q}=(0.5696, 0.8608)$$

the sample covariance matrix is calculated to be

$$S_{q}=\left(\begin{array}{ll} 0.0270 & 0.0067\\ 0.0067 & 0.0074 \end{array}\right),$$

and the sample correlation is calculated to be $\rho_{q}=0.4771$. We observe a moderate positive association between the parameters $q_{1}$ and $q_{2}$, while in the previous example, we observed a moderate negative association between the parameters. The statistics obtained in the second example (i.e. using the WrisTAS*^™^*7 biosensor) may not be comparable to the statistics obtained in the first example (i.e. using the SCRAM biosensor) due to the fact that the two sets of 9 drinking episodes are not identical; in other words, we lack data gathered by the two biosensors in identical conditions to eliminate the potential confounding factors. In addition, the sample size of 9 drinking episodes is a small sample size to make any general hypothesis regarding the correlation between the two parameters $q_{1}$ and $q_{2}$.

Tables 7–9 display these statistics along with the measured (or actual) value obtained by the WrisTAS*^™^*7 alcohol biosensor as well as the conservative 95% error band for the 9 drinking episodes from the testing set. From these tables, we can observe that the 95% error bands do a reasonably good job of capturing the actual values of these statistics specially for the value of the peak TAC and the area under the curve displayed in Table 7 and 9. In Figure 5, we can see that there are minor fluctuations in the measured TAC curve. Similar to the previous example, if we smooth the measured TAC curve, we will obtain better results in lowering the error between the time at which the peak value of the TAC is attained using the smoothed TAC curve and the estimated peak time compared to the results obtained in Table 8. The smoothed version results are included in Table 11 in the Supplementary Section 9.

## 8. Discussion and Concluding Remarks

In this paper, we considered the nonparametric fitting of a population model for the transdermal transport of alcohol based on a maximum likelihood approach applied to a mixed effects statistical model. In estimating a population model, we were actually estimating the distribution of the model parameters and consequently the MLE problem was formulated as an optimization problem over a space of feasible probability measures endowed with the weak topology induced by the Prohorov metric. A notable advantage of our developed methodology is that by considering a nonparametric approach, there is no need to make any assumptions about the distribution being estimated.

By using a first principles physics based model in the form of a one dimensional diffusion equation, we were able to capture the essential features of transdermal transport while keeping the dimension of the parameter space low. In this way, we were able to avoid having to introduce regularization so as to mitigate ill-posedness and over-fitting. On the other hand, the fact that the model was infinite dimensional being based on a partial differential equation, computing the MLE necessitated finite dimensional approximation.

We were able to first theoretically demonstrate the existence and then the consistency of our MLE using a decades old result from the literature. The consistency result is with respect to the uncertainty across subjects. It is likely that the consistency results proved in [22] and [21], in the context of a naive pooled statistical model based on a nonlinear least squares estimator, for problems either the same as, or very similar to the one we consider here, would apply for the uncertainty within each subject (i.e. as the resolution of the data with respect to time increases). At present, this is just a hypothesis and a possible avenue for future research; as of yet, we have not carefully examined this possibility.

In addition, we were able to use linear semigroup theory, in particular the Trotter-Kato Theorem, and the properties of the weak topology and the Prohorov metric on the space of feasible probability measures, to establish a convergence result with respect to the MLE for the finite dimensional approximating estimation problems and the MLE for the estimation problem posed in terms of the original underlying infinite dimensional model.

We were able to demonstrate the efficacy of our theoretical results numerically first on an example involving simulated data and then on one involving actual human subject data from an NIH funded study. We used our scheme to obtain the joint density and distribution of the parameters as well as estimates and conservative 95% error bands for the TAC signal and a number of TAC related statistics of particular interest to researchers and clinicians who work in the area of alcohol use disorder.

A similar methodology has been developed in [47] using a method called the Nonparametric Adaptive Grid (NPAG) algorithm. In their approach, the grid of support points is iteratively changed in the algorithm which may add to the computational cost, and also as mentioned in [47], condensing the grid around the nodes with the highest probability may result in estimating only a local maximum likelihood which may not capture the “true” distribution to be estimated in case of a multimodal distribution. One additional difference is that their method is tested on simulated data using a normal distribution for the parameters and it has not been tested on a human subject data. Instead of condensing the grid, we use a fixed grid and directly maximized the log-likelihood function using a mixed-effects model which was tested on both simulated data and on human subject data.

In addition to consistency with respect to the intrinsic uncertainty, other extensions we are currently looking at include the development of a general framework for estimating random parameters in general finite or infinite dimensional, continuous or discrete-time dynamical systems (e.g. ODEs (ordinary differential equations), PDEs (partial differential equations), FDEs (functional differential equations), DEs (difference equations), etc.) that would potentially subsume the results presented here as well as in [22] and [21].

Finally, although correlated, TAC and BrAC are not quantitatively equivalent. BrAC is a good indicator of BAC, which is an informative quantitative measure of the level of alcohol in the body at that moment that is affecting judgment/decision-making, motor coordination, and cognition. On the other hand, TAC is temporally delayed from BrAC since it must pass through the skin prior to being measured, and TAC levels vary across individuals and devices and within and between drinking episodes, unlike BrAC which is relatively consistent across people and conditions. This makes TAC difficult to interpret in relation to alcohol levels in the blood at a given timepoint, limiting its usefulness as a quantitative measure of alcohol in the body. Thus, having a way to estimate BrAC from TAC would increase its utility for researchers, clinicians, and individuals who want quantitatively and temporally meaningful information about alcohol levels in the body.

Since the actual motivation for this investigation is the development of schemes for converting biosensor measured TAC into BAC/BrAC, the next step would be to examine how well population models, estimated using the approach we have presented here, perform when used as part of a scheme that deconvolves an estimate for BAC/BrAC from the TAC signal. In particular, we are interested in comparing it to the schemes, used for this same purpose, developed and implemented in [11,12,28–31]. In addition, we are also interested in examining how our uncertainty quantification scheme for the TAC to BAC/BrAC conversion problem performs when compared to the non-physics based, machine learning inspired schemes developed in [18–20, 34].

## Supplementary Material

1

## Figures and Tables

**Table 1.** Decrease in D, the sum of the squared differences at each node between the estimated and the “true” distribution, with increasing m, the number of drinking episodes, for fixed values of the number of nodes M=400 and the level of discretization N=128.

m	D
1	39.0164
3	28.3091
7	8.3247
9	7.1750
16	3.5697
42	3.0337

**Table 2.** Decrease in ${\overset{‾}{D}}_{M}$, the normalized sum of the squared differences at each node between the estimated distribution and the “true” distribution, with increasing M, the number of nodes, for a fixed value for the level of discretization, N=128.

M	${\overset{‾}{D}}_{M}$
25	0.03795
100	0.03025
225	0.02974
400	0.02081

**Table 3.** Decrease in ${\overset{‾}{D}}_{N}$, the normalized sum of the squared differences at each node between the estimated distribution and the “true” distribution, with increasing N, the level of discretization, with the number of nodes fixed at M=400.

N	${\overset{‾}{D}}_{N}$
4	1.22783
16	0.65644
64	0.18565
128	0.06504

**Table 4.** The measured peak TAC, estimated peak TAC, and the 95% error band for the 9 drinking episodes from the testing set collected using the SCRAM alcohol biosensor.

Drinking Episode	Measured Peak TAC	Estimated Peak TAC	95% Error Band
1	0.0585	0.0413	(0.0327, 0.0539)
2	0.0477	0.0433	(0.0324, 0.0543)
3	0.0464	0.0391	(0.0320, 0.0501)
4	0.0417	0.0375	(0.0301, 0.0489)
5	0.0535	0.0618	(0.0497, 0.0781)
6	0.0419	0.0361	(0.0287, 0.0465)
7	0.0450	0.0441	(0.0346, 0.0558)
8	0.0405	0.0430	(0.0345, 0.0542)
9	0.0391	0.0402	(0.0313, 0.0508)

**Table 5.** The measured peak time, estimated peak time, and the 95% error band for the 9 drinking episodes from the testing set collected using the SCRAM alcohol biosensor.

Drinking Episode	Measured Peak Time	Estimated Peak Time	95% Error Band
1	2.5600	2.4480	(1.9200, 2.8800)
2	2.2400	2.6080	(2.2400, 2.8800)
3	2.2400	2.8928	(2.5600, 3.2000)
4	4.1600	2.9056	(2.5600, 3.2000)
5	2.2400	2.5728	(2.2400, 2.8800)
6	2.2400	2.8928	(2.5600, 3.2000)
7	3.2000	2.9696	(2.5600, 3.2000)
8	2.8800	2.9312	(2.5600, 3.2000)
9	3.2000	2.9088	(2.5600, 3.2000)

**Table 6.** The measured area under the curve (AUC), estimated AUC, and 95% error band for 9 drinking episodes from the testing set collected using the SCRAM alcohol biosensor.

Drinking Episode	Measured AUC	Estimated AUC	95% Error Band
1	0.1909	0.1784	(0.1382, 0.2229)
2	0.1876	0.1799	(0.1343, 0.2321)
3	0.1868	0.1751	(0.1364, 0.2421)
4	0.1765	0.1735	(0.1349, 0.2394)
5	0.2231	0.2963	(0.2203, 0.3911)
6	0.1151	0.1496	(0.1117, 0.1982)
7	0.1474	0.1912	(0.1444, 0.2563)
8	0.1277	0.1898	(0.1412, 0.2505)
9	0.1493	0.1750	(0.1307, 0.2319)

**Table 7.** The measured peak TAC, estimated peak TAC, and the 95% error band for the 9 drinking episodes from the testing set collected using the WrisTAS*^™^*7 alcohol biosensor.

Drinking Episode	Measured Peak TAC	Estimated Peak TAC	95% Error Band
1	0.0228	0.0265	(0.0215, 0.0336)
2	0.0259	0.0258	(0.0221, 0.0319)
3	0.0270	0.0218	(0.0195, 0.0266)
4	0.0222	0.0212	(0.0173, 0.0263)
5	0.0249	0.0205	(0.0168, 0.0264)
6	0.0276	0.0263	(0.0221, 0.0337)
7	0.0287	0.0276	(0.0228, 0.0348)
8	0.0182	0.0236	(0.0197, 0.0286)
9	0.0284	0.0296	(0.0247, 0.0371)

**Table 8.** The measured peak time, estimated peak time, and the 95% error band for the 9 drinking episodes from the testing set collected using the WrisTAS*^™^*7 alcohol biosensor.

Drinking Episode	Measured Peak Time	Estimated Peak Time	95% Error Band
1	2.1667	2.2758	(1.9167, 2.6667)
2	1.6000	3.0125	(2.8333, 3.5000)
3	1.6944	2.5542	(2.0833, 3.3333)
4	2.4167	2.5083	(2.3333, 2.9167)
5	2.4167	2.3117	(2.1229, 2.5833)
6	2.4167	2.6917	(2.5000, 3.0000)
7	1.7500	2.3358	(2.0833, 2.8333)
8	2.5833	1.9383	(1.8333, 2.0833)
9	1.5208	2.6233	(2.3333, 3.0833)

**Table 9.** The measured area under the curve (AUC), estimated AUC, and 95% error band for 9 drinking episodes from the testing set collected using the WrisTAS*^™^*7 alcohol biosensor.

Drinking Episode	Measured AUC	Estimated AUC	95% Error Band
1	0.0755	0.1054	(0.0912, 0.1229)
2	0.0937	0.1205	(0.1057, 0.1438)
3	0.1091	0.1022	(0.0927, 0.1240)
4	0.0653	0.0876	(0.0759, 0.1023)
5	0.0902	0.0730	(0.0647, 0.0871)
6	0.0942	0.1044	(0.0917, 0.1248)
7	0.1036	0.1213	(0.1063, 0.1448)
8	0.0716	0.0876	(0.0760, 0.1038)
9	0.0942	0.1298	(0.1138, 0.1549)
