# Supplementary material for: Computation of Nonparametric, Mixed Effects, Maximum Likelihood, Biosensor Data Based-Estimators for the Distributions of Random Parameters in an Abstract Parabolic Model for the Transdermal Transport of Alcohol
Source: Math Biosci Eng. Author manuscript; Available in PMC 2024 Dec 2. (PMC11610795; doi:10.3934/mbe.2023900)
Supplement: 1 [file NIHMS2023189-supplement-1.pdf]

9. Supplementary

As mentioned in Section 7.2, smoothing the measured TAC curve results in lowering the error between the time at which the peak value of the TAC is attained using the smoothed TAC curve and the estimated peak time. Here, we include the results using the smoothed version of the TAC curve. The following Tables 10 and 11 correspond to Tables 5 and 8, respectively, with the smoothed TAC curve used instead of the measured TAC curve.

Table 10. The peak time using the smoothed TAC curve, estimated peak time, and the 95% error band for the 9 drinking episodes from the testing set collected using the SCRAM alcohol biosensor.

Drinking Episode	Smoothed Peak Time	Estimated Peak Time	95% Error Band
1	3.2000	2.4480	(1.9200, 2.8800)
2	2.8800	2.6080	(2.2400, 2.8800)
3	2.8800	2.8928	(2.5600, 3.2000)
4	3.8400	2.9056	(2.5600, 3.2000)
5	3.2000	2.5728	(2.2400, 2.8800)
6	2.8800	2.8928	(2.5600, 3.2000)
7	3.5200	2.9696	(2.5600, 3.2000)
8	3.5200	2.9312	(2.5600, 3.2000)
9	3.2000	2.9088	(2.5600, 3.2000)

Table 11. The peak time using the smoothed TAC curve, estimated peak time, and the 95% error band for the 9 drinking episodes from the testing set collected using the WrisTAS^{TM}7 alcohol biosensor.

Drinking Episode	Smoothed Peak Time	Estimated Peak Time	95% Error Band
1	2.6667	2.2758	(1.9167, 2.6667)
2	2.1667	3.0125	(2.8333, 3.5000)
3	2.3333	2.5542	(2.0833, 3.3333)
4	2.9167	2.5083	(2.3333, 2.9167)
5	2.6667	2.3117	(2.1229, 2.5833)
6	2.6667	2.6917	(2.5000, 3.0000)
7	2.0833	2.3358	(2.0833, 2.8333)
8	2.6667	1.9383	(1.8333, 2.0833)
9	2.0833	2.6233	(2.3333, 3.0833)

To smooth the TAC curve, we used cubic splines and we measured the discrepancy between the

smoothed peak time and the estimated peak time using the mean squared error. For the SCRAM biosensor, the mean squared error decreased from 0.314 (prior to smoothing) to 0.293 (after smoothing). For the WrisTAS^{TM}7 biosensor, the mean squared error decreased from 0.535 (prior to smoothing) to 0.233 (after smoothing).
